# The persistence of low-grade inflammatory monocytes contributes to aggravated atherosclerosis

**DOI:** 10.1038/ncomms13436

**Published:** 2016-11-08

**Authors:** Shuo Geng, Keqiang Chen, Ruoxi Yuan, Liang Peng, Urmila Maitra, Na Diao, Chun Chen, Yao Zhang, Yuan Hu, Chen-Feng Qi, Susan Pierce, Wenhua Ling, Huabao Xiong, Liwu Li

**Affiliations:** 1 Department of Biological Sciences, Virginia Tech, Blacksburg, Virginia 24061-0910, USA; 2 Department of Medicine, Immunology Institute, Icahn School of Medicine at Mount Sinai, New York, New York 10029, USA; 3 Laboratory of Immunogenetics, National Institute of Allergy and Infectious Diseases, National Institutes of Health, Bethesda, Maryland 20892, USA; 4 School of Public Health, National Sun Yat-Sen University, Guangzhou 510080, China

## Abstract

Sustained low-grade inflammation mediated by non-resolving inflammatory monocytes has long been suspected in the pathogenesis of atherosclerosis; however, the molecular mechanisms responsible for the sustainment of non-resolving inflammatory monocytes during atherosclerosis are poorly understood. Here we observe that subclinical endotoxemia, often seen in humans with chronic inflammation, aggravates murine atherosclerosis through programming monocytes into a non-resolving inflammatory state with elevated Ly6C, CCR5, MCP-1 and reduced SR-B1. The sustainment of inflammatory monocytes is due to the disruption of homeostatic tolerance through the elevation of miR-24 and reduction of the key negative-feedback regulator IRAK-M. miR-24 reduces the levels of Smad4 required for the expression of IRAK-M and also downregulates key lipid-processing molecule SR-B1. IRAK-M deficiency in turn leads to elevated miR-24 levels, sustains disruption of monocyte homeostasis and aggravates atherosclerosis. Our data define an integrated feedback circuit in monocytes and its disruption may lead to non-resolving low-grade inflammation conducive to atherosclerosis.

## Abstract (web summary)

Chronic low-grade inflammation has been suspected to promote atherosclerosis. Here, Geng *et al*. show that sustained low-grade inflammation promotes atherosclerosis in mice via monocyte programing that involves a coupled disruption of IRAK-M regulation and induction of miR-24.

## Graphical abstract

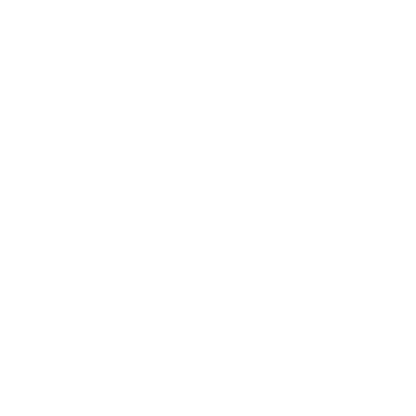

Atherosclerosis and related cardiovascular complications are among the leading causes of morbidity and mortality in industrialized countries^1^. Complex and intertwined alterations in non-resolving low-grade inflammation and lipid metabolism may collectively contribute to the initiation and progression of atherosclerosis. Despite growing appreciation of low-grade inflammation and its correlation with the complex pathogenesis of atherosclerosis, the fundamental mechanisms responsible for the establishment of non-resolving low-grade inflammation are not well understood and present a major impediment for the development of effective cures.

At the cellular level, innate monocyte is one of the key linchpins that connect low-grade inflammation and altered lipid metabolism, through the expression of inflammatory mediators as well as the modulation of intra-cellular lipid accumulation^2^. Inflammatory mediators are critically involved in the coordination of atherosclerotic plaque composition and stability, whereas lipid deposition inside infiltrating monocytes/macrophages may lead to the generation of foam cells within the atherosclerotic core^3^. Differential activation states of monocytes may govern the expression profiles of inflammatory mediators and the surface levels of lipid transporters such as SR-B1. In the context of resolving inflammation, the expression of pro-inflammatory mediators by inflammatory monocytes is transient and subsequently suppressed due to the induction of homeostatic negative regulators, a phenomenon exemplified in endotoxin tolerance^4^. In contrast, under non-resolving inflammatory conditions, it has been suggested that monocytes may fail to develop endotoxin tolerance and adopt a sustained inflammatory state conducive for the pathogenesis of atherosclerosis^5^. Despite its significance, causes and molecular mechanisms responsible for the disruption of tolerance and non-resolving inflammatory monocyte polarization conducive for the pathogenesis of atherosclerosis are not well understood.

Clinical risk factors for non-resolving inflammation and atherosclerosis such as chronic infection, obesity and ageing often lead to mucosal leakages and subclinical levels of circulating bacteria endotoxin liposaccharide (LPS)^6^,^7^. Recent clinical studies suggest that subclinical endotoxemia may be closely correlated with the occurrence of chronic low-grade inflammatory diseases in humans and animal models such as atherosclerosis^8^,^9^. Studies conducted *in vitro* suggest that subclinical super-low levels of endotoxin fail to cause compensatory endotoxin tolerance and may instead be capable of inducing low-grade inflammatory responses from cultured monocytes/macrophages^10^,^11^. However, *in vivo* study is lacking to examine the monocyte polarization by subclinical endotoxemia and its pathological consequences in atherosclerosis.

At the molecular level, we reported that the reduction of homeostatic negative regulators necessary for causing tolerance and preventing runaway inflammation may be a potential cause for low-grade inflammatory polarization of macrophages *in vitro*^11^,^12^. Subclinical levels of endotoxin may reduce negative regulators such as nuclear receptors, Tollip and interleukin-1 receptor associated kinase M (IRAK-M) known to be critical for resolving inflammation and homeostatic tolerance^13^,^14^,^15^. Of particular interest, IRAK-M is a key negative regulator of innate immunity signalling pathways within monocytes/macrophages, through inhibiting the activation of mitogen-activated protein kinases and subsequent induction of inflammatory cytokines^13^,^16^. The expression of IRAK-M mediated through Smad4 under resolving inflammatory conditions is critically responsible for monocyte homeostatic tolerance^17^,^18^,^19^,^20^,^21^,^22^. In sharp contrast, we reported that monocytes challenged with subclinical super-low-dose LPS *in vitro* have reduced levels of IRAK-M, with unknown mechanism^11^.

In this current study, we aim to test the pathological consequence of subclinical endotoxemia during the polarization of low-grade inflammatory monocytes and progression of atherosclerosis. We hypothesize that the reduction of IRAK-M by low-grade inflammation may set in motion a sustained programme of inflammatory polarization and altered lipid deposition in monocytes, which may contribute to the aggravation of atherosclerosis. Our data uncovered a coupled disruption of IRAK-M regulation and induction of miR-24 that collectively disrupt homeostatic tolerance, thereby sustaining the non-resolving low-grade inflammatory monocyte phenotype, conducive for the aggravation of atherosclerosis.

## Results

### Low-grade inflammation aggravates atherosclerosis

**Figure 1:**
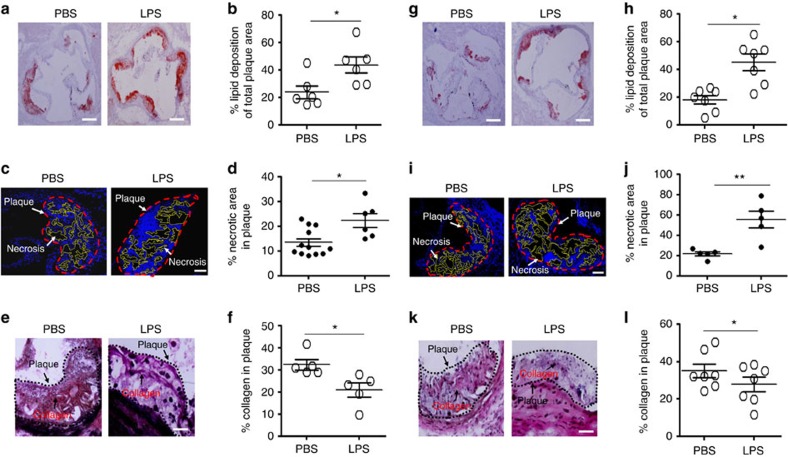
Subclinical endotoxemia aggravates the progression of atherosclerosis. (**a**–**f**) *ApoE*^−/−^ mice were conditioned with PBS or super-low-dose LPS together with HFD for 8 weeks. (**a**) Representative images of atherosclerotic plaques within aortic root areas stained by Oil Red O. Scale bar, 300 μm. (**b**) Ratios of the lipid deposition area among the total atherosclerotic plaque area within the aortic root from PBS mice (*n*=6) and super-low-dose LPS-conditioned mice (*n*=6). (**c**) Representative images of necrotic core areas within the aortic roots. Scale bar, 100 μm. (**d**) Ratios of the necrotic area among the total atherosclerotic plaque area from PBS (*n*=12) and super-low-dose LPS-conditioned (*n*=6) mice. (**e**) Representative images of collagen containing areas within aortic roots. Scale bar, 100 μm. (**f**) Ratios of the collagen-containing area among the total atherosclerotic plaque area within the aortic root from PBS (*n*=5) and super-low-dose LPS-conditioned (*n*=5) mice. (**g**–**l**) *ApoE*^−/−^ mice were pre-conditioned with PBS or super-low-dose LPS for 4 weeks together with HFD, followed by HFD feeding only for an additional 4 weeks. (**g**) Representative images of atherosclerotic plaques within aortic root areas stained by Oil Red O. Scale bar, 300 μm. (**h**) Quantification of the lipid deposition area as a percentage of the total atherosclerotic plaque area within the aortic root from PBS (*n*=7) and super-low-dose LPS-conditioned (*n*=7) mice. (**i**) Representative images of necrotic core areas within the aortic roots. Scale bar, 100 μm. (**j**) Ratios of the necrotic area among the total atherosclerotic plaque area within the aortic root from PBS (*n*=5) and super-low-dose LPS-conditioned (*n*=5) mice. (**k**) Representative images of collagen containing areas within aortic roots. Scale bar, 100 μm. (**l**) Ratios of the collagen containing area among the total atherosclerotic plaque area within the aortic root from PBS (*n*=7) and super-low-dose LPS-conditioned (*n*=7) mice. Error bars show means±s.e.m. All data are representative of at least two similar experiments. *P<0.05 and **P<0.01; Student's *t*-test.

Although sustained low-grade inflammation has long been suspected in the pathogenesis of atherosclerosis, the exact role of low-grade inflammation is still not clearly defined^23^. To explore the contribution of low-grade inflammation to the development of atherosclerosis, we first tested the effect of subclinical super-low-dose LPS on the pathogenesis of atherosclerosis in the Apolipoprotein E-deficient animal model. As determined by Oil Red O staining of the aorta sections, chronic injection of super-low-dose LPS significantly elevated the lipid deposition ratio within the atherosclerotic plaques in ApoE-deficient mice fed with the western high-fat diet (HFD) for 8 weeks, as compared with mice fed with HFD alone (Fig. 1a,b). We further documented significantly elevated areas of the necrotic cores within the plaques (Fig. 1c,d). Immunohistochemical staining revealed a reduction in collagen content of the plaques (Fig. 1e,f). Collectively, these data suggest that subclinical dose LPS exacerbates the pathogenesis of atherosclerosis in mice.

Considering the possible involvement of innate immune memory^24^, we further tested whether a shorter period of super-low-dose LPS pre-conditioning may have a lasting impact and be sufficient to aggravate HFD-associated atherosclerosis. To test that, we pre-conditioned HFD-fed *ApoE*^−/−^ mice with super-low-dose LPS injection for 4 weeks, followed by continued HFD feeding for an additional 4 weeks without further LPS injection. We observed that a short-time LPS pre-conditioning caused similar impacts by elevating lipid content within atherosclerotic plaques, increasing sizes of necrotic cores and reducing plaque contents of collagens and smooth muscle actin (Fig. 1g–l and Supplementary Fig. 1). Based on these observation, we focused our subsequent analyses with the short LPS pre-conditioning model.

**Figure 2:**
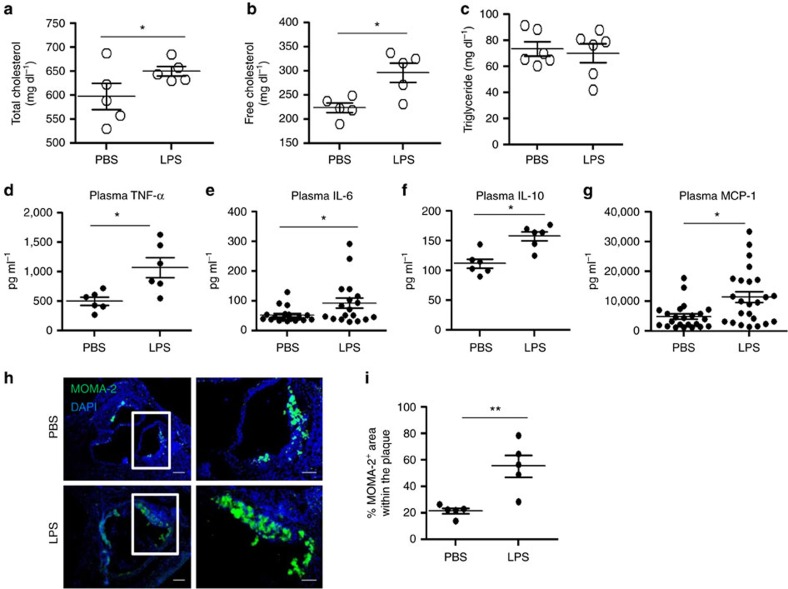
Subclinical endotoxemia induces low-grade inflammation *in vivo*. *ApoE*^−/−^ mice were pre-conditioned with PBS or super-low-dose LPS for 4 weeks together with HFD, followed by HFD feeding only for an additional 4 weeks. The levels of total (**a**) and free cholesterol (**b**) in the plasma were tested. Data are shown from PBS (*n*=5) and super-low-dose LPS-conditioned (*n*=5) mice. (**c**) Triglyceride levels in the plasma from PBS (*n*=6) and super-low-dose LPS-conditioned (*n*=6) mice. (**d**) Plasma tumour necrosis factor (TNF)-α levels from PBS (*n*=6) and super-low-dose LPS-conditioned (*n*=6) mice. (**e**) Plasma IL-6 levels from PBS (*n*=18) and super-low-dose LPS-conditioned (*n*=18) mice. (**f**) Plasma IL-10 levels from PBS (*n*=5) and super-low-dose LPS-conditioned (*n*=6) mice (left panel). (**g**) Plasma MCP-1 levels from PBS (*n*=23) and super-low-dose LPS-conditioned (*n*=24) mice (right panel). (**h**) Representative images of MOMA-2^+^ macrophages in the atherosclerotic plaques of aortic root areas. Scale bar, left panel: 300 μm; right panel: 100 μm. (**i**) Quantification of MOMA-2^+^ area as a percentage of the total atherosclerotic plaque area within the aortic root. Data are shown for aortic plaque areas from PBS (*n*=5) and super-low-dose LPS-conditioned (*n*=5) mice. Data are representative of two similar experiments. *P<0.05 and **P<0.01; Student's *t*-test.

We further examined the levels of selected inflammatory mediators and plasma lipids in ApoE-deficient mice fed with HFD, injected with either PBS or super-low-dose LPS. Super-low-dose LPS injection caused significant elevation of plasma levels of total cholesterol and free cholesterol, without affecting the levels of triglycerides (Fig. 2a–c). In terms of inflammatory mediators, super-low-dose LPS caused significant elevation of pro-inflammatory cytokines such as tumour necrosis factor-α and interleukin (IL)-6, as well as chemokine MCP-1 (Fig. 2d–g).

Given the elevated levels of MCP-1, we further tested whether there were increased levels of tissue macrophages within the atherosclerotic plaques. Indeed, immunohistochemical staining revealed significantly elevated levels of macrophages within the plaque areas of HFD-fed ApoE-deficient mice injected with super-low-dose LPS as compared with PBS controls (Fig. 2h–i). Our data indicate that super-low-dose LPS conditioning contributed to elevated inflammation, increased recruitment of macrophages, as well as elevated plasma cholesterol contents.

### Polarized monocytes aggravate atherosclerosis

**Figure 3:**
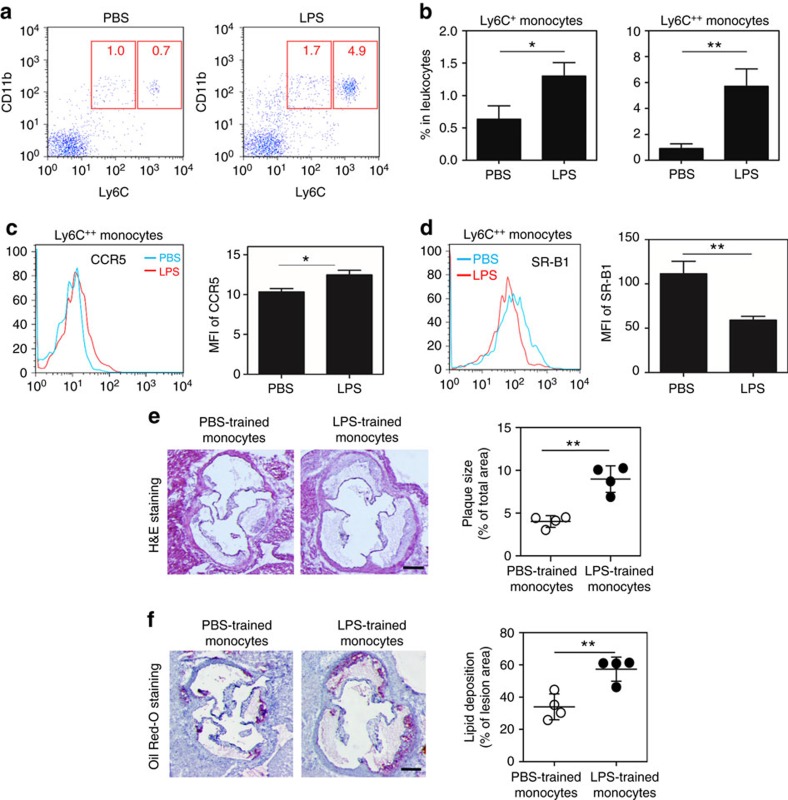
Polarized inflammatory monocytes by subclinical endotoxin contributes to aggravated atherosclerosis. *ApoE*^−/−^ mice were pre-conditioned with PBS or super-low-dose LPS for 4 weeks together with HFD, followed by HFD feeding only for an additional 4 weeks. (**a**) Peripheral blood cells were collected, and CD11b^+^Ly6C^++^ and CD11b^+^Ly6C^+^ monocytes gated within the Ly6G^−^ population were examined by flow cytometry. (**b**) The frequency of inflammatory monocytes within total leukocytes was quantified. Data are shown from PBS (*n*=7) and super-low-dose LPS-conditioned (*n*=7) mice. (**c**) The expression levels of CCR5 within circulating inflammatory monocytes were compared between PBS (*n*=6) and LPS-conditioned (*n*=6) mice. (**d**) The expression levels of SR-B1 within circulating inflammatory monocytes were compared between PBS (*n*=6) and LPS conditioned (*n*=6) mice. (**e**,**f**) Adoptive transfer of LPS-programmed monocytes exacerbates atherosclerosis. BM cells from *ApoE*^−/−^ mice were cultured with M-CSF (10 ng ml^−1^) in the presence of PBS or LPS (100 pg ml^−1^) for 5 days. PBS- or LPS-programmed BM cells (3 × 10^6^ cells per mouse) were then adoptively transferred through intravenous injection to HFD-fed *ApoE*^−/−^ mice once a week for 4 weeks. (**e**) Representative images of atherosclerotic plaques within aortic root areas stained by haematoxylin and eosin. Plaque sizes were quantitated as a percentage of the lesion areas within aortic root areas. Scale bar, 300 μm. (**f**) Representative images of atherosclerotic plaques within aortic root areas stained by Oil Red O. Scale bar, 300 μm. The lipid deposition areas as a percentage of the total atherosclerotic plaques within the aortic root areas were quantified. Data are presentative of two similar experiments. Error bars represent means±s.e.m.; *P<0.05 and **P<0.01; Student's *t*-test.

Innate monocytes/macrophages are key factors that contribute to the expression of inflammatory mediators and altered lipid metabolism, and play critical roles during the pathogenesis of atherosclerosis^5^. To test whether aggravated atherosclerosis in mice conditioned with super-low-dose LPS may be due to distinct alterations in monocytes/macrophages, we examined the activation status of circulating monocytes and macrophages within atherosclerotic plaques. We observed that HFD-fed ApoE-deficient mice conditioned with super-low-dose LPS had significantly higher levels of circulating CD11b^+^Ly6C^Positive^ pro-inflammatory monocytes as compared with mice conditioned with PBS (Fig. 3a,b). In addition, the levels of inflammatory chemotaxis receptor CCR5, a key monocyte marker associated with atherosclerosis^25^, were significantly increased on circulating monocytes from HFD-fed *ApoE*^−/−^ mice conditioned with super-low-dose LPS (Fig. 3c). We next examined the cell surface levels of SR-B1, a key modulator of monocyte inflammation and lipid metabolism^26^,^27^. Flow cytometry analyses revealed that SR-B1 expression levels were significantly reduced on circulating monocytes from HFD-fed *ApoE*^−/−^ mice conditioned with super-low-dose LPS as compared with mice conditioned with PBS (Fig. 3d). Similar reduction in SR-B1 levels were also observed in elutriated monocytes within the aorta of HFD-fed mice conditioned with super-low-dose LPS as compared with the aorta from mice conditioned with PBS (Supplementary Fig. 2). Immunohistochemical staining of atherosclerotic plaque areas corroborated with flow analyses and revealed significantly reduced levels of SR-B1 within Monomac-positive plaque macrophages (Supplementary Fig. 2). On the other hand, the levels of SR-A within Monomac-positive plaque macrophages from HFD-fed ApoE-deficient mice conditioned with super-low-dose LPS were significantly elevated (Supplementary Fig. 2), consistent with elevated inflammatory status of infiltrating macrophages as reported in other systems^28^.

Our data revealed the unique programming of low-grade inflammatory monocytes by super-low-dose LPS and suggest that programmed inflammatory monocytes may be responsible for aggravated atherosclerosis in mice injected with super-low-dose LPS. As systemic LPS injection may not only affect monocyte programming, but also other tissues such as the liver and metabolic processes^29^,^30^, we further examined whether the re-programmed monocytes, instead of other systemic effects of LPS, may be causally involved in the exacerbation of atherosclerosis. To address this issue, we performed adoptive transfer experiment with *in vitro* programmed monocytes. Bone-marrow monocytes harvested from *ApoE*^−/−^ mice were programmed *in vitro* by supplementing the culture with either subclinical dose LPS (100 pg ml^−1^) or PBS for 5 days^31^. *In vitro* cultured monocytes with subclinical dose LPS exhibit characteristic phenotype as compared with monocytes programmed *in vivo* through LPS injection, as characterized by elevated levels of CCR5 and reduced levels of SR-B1 (Supplementary Fig. 3).

*In vitro* programmed monocytes by either PBS or LPS were subsequently washed with LPS-free and sterile PBS, and intravenously injected into HFD-fed *ApoE*^−/−^ mice once weekly for 1 month. At the end of the adoptive transfer regimen, we observed significant increases in the plaque sizes as measured by haematoxylin and eosin staining, as well as Oil Red O staining in mice transfused with LPS-programmed monocytes as compared with mice transfused with PBS-programmed monocytes (Fig. 3e,f). In contrast to LPS injection, which also caused alterations in plasma lipids, the overall levels of plasma lipids, body weights and liver expression levels of SR-B1 were not altered among *ApoE*^−/−^ mice transfused with either PBS-programmed or LPS-programmed monocytes (Supplementary Fig. 3). Our data indicate that programmed monocytes by subclinical dose LPS can directly contribute to the aggravation of atherosclerosis progression.

### Polarized monocytes reduce SR-B1 expression through miR-24

**Figure 4:**
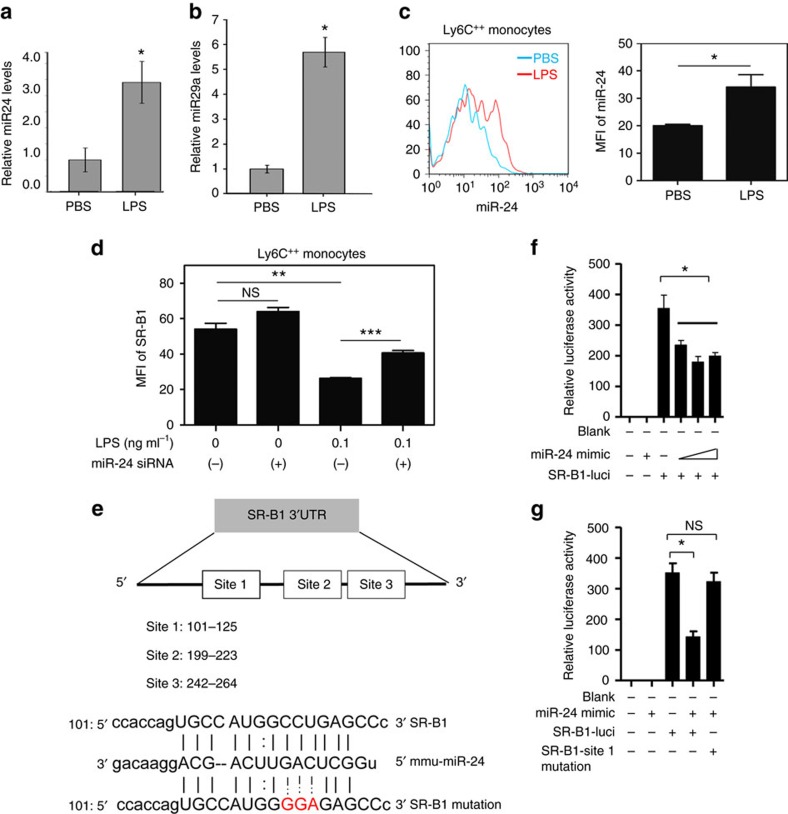
Suppression of SR-B1 in inflammatory monocytes by super-low-dose LPS is dependent on miR-24 induction. (**a**,**b**) *ApoE*^−/−^ mice were pre-conditioned with PBS or super-low-dose LPS for 4 weeks together with HFD, followed by HFD feeding only for an additional 4 weeks. Total miRs isolated from splenocytes were used for real-time reversre transcriptase–PCR analyses for the relative levels of miR-24 (**a**) and miR-29 (**b**). (**c**) BMMs from C57 BL/6 mice were cultured with M-CSF (10 ng ml^−1^) in the presence super-low-dose LPS (0.1 ng ml^−1^) for 3 days and fresh LPS was added to the cell cultures every 2 days. Fluorescent RNA probe for miR-24-3p was added to the cell cultures 16 h before harvesting. The expression levels of miR-24-3p within CD11b^+^Ly6C^++^ monocytes were examined by flow cytometry. (**d**) BMMs from C57 BL/6 mice were cultured with M-CSF (10 ng ml^−1^) in the presence super-low-dose LPS (0.1 ng ml^−1^) and miR-24 antagomir was added to indicated cultures. Fresh LPS and antagomir was added to the cell cultures every 2 days. On day 5, cells were harvested and expression levels of SR-B1 within CD11b^+^Ly6C^++^ monocytes were analysed by flow cytometry. (**e**) A schematic illustration of the SR-B1 3′-UTR and potential miR-24-binding sites. (**f**,**g**) Luciferase activity assays in 293T cells transfected with either WT or mutant SR-B1 3′-UTR luciferase reporter plasmids in the presence of miR-24 mimic or scrambles. Quantified data are shown from cells with indicated treatment (*n*=3) and results are representative of three experiments. Error bars show means±s.e.m.; NS, not significant; *P<0.05, **P<0.01 and ***P<0.001; (**a**,**b**,**c**) Student's *t*-test; (**d**,**f**,**g**) one-way analysis of variance.

Although the low-grade inflammatory monocyte phenotype programmed by super-low dose LPS may bear critical pathological relevance, the underlying mechanisms are poorly studied nor understood. Almost all past studies regarding LPS used much higher dosages of LPS, which were recently shown to illicit TLR4-independent toxicity effects through intra-cellular association with caspases or other molecules^32^,^33^,^34^. In contrast, super-low-dose LPS that we used represent pathologically relevant dosages seen in humans and experimental animals with chronic diseases^35^,^36^. We also reported that super-low-dose LPS critically requires cell-surface TLR4 for signalling^11^. To understand the mechanisms responsible for the polarization of inflammatory monocytes conditioned by super-low-dose LPS, we performed microRNA sequencing (miRNAseq) analyses of cultured bone marrow monocytes/macrophages (BMMs). Induced miRNAs including miR-24 and miR-29 by super-low-dose LPS identified through miRNAseq were listed in Supplementary Table 1. We further confirmed the elevated expression of miR-24 and miR-29 in HFD-fed ApoE-deficient mice conditioned with super-low-dose LPS as compared with PBS-conditioned control mice (Fig. 4a,b). miR-29 was known to have potent inhibitory effects on collagens and the transforming growth factor-β signalling pathway^37^. Thus, the induction of miR-29 by super-low-dose LPS correlates well with the reduced plaque collagen content in mice conditioned with super-low-dose LPS *in vivo*.

We focused our attention to examine the pathophysiological relevance of miR-24 induction by super-low-dose LPS, as miR-24 is among the most highly expressed miRNAs previously observed in both human patients with familial hypercholesterolemia, as well as in HFD-fed ApoE-deficient mice^38^. A previous animal study also suggests that miR-24 may be correlated with lipid accumulation and hyperlipidemia^39^. Through flow cytometry analyses, we observed that super-low-dose LPS selectively induced the expression levels of miR-24 in the CD11b^+^Ly6C^++^ pro-inflammatory monocytes (Fig. 4c). Application of miR-24 antagomir in cultured BMM restored the expression levels of SR-B1 (Fig. 4d). We next searched the 3′-untranslated region ( 3′-UTR) of SR-B1 and found putative miR-24-binding sites (Fig. 4e). Luciferase reporter assays demonstrated that miR-24 dose dependently reduced the SR-B1 target messenger RNA stability (Fig. 4f). In contrast, miR-24 mimic failed to exert its degradation effect on mutant SR-B1 target with defective miR-24-binding site (Fig. 4g). RNA co-immunoprecipitation analyses showed direct association of miR-24 with the SR-B1 3′-UTR within the microprocessor complex (Supplementary Fig. 4).

### Polarization of monocytes through reduction of Smad-4 and IRAK-M

**Figure 5:**
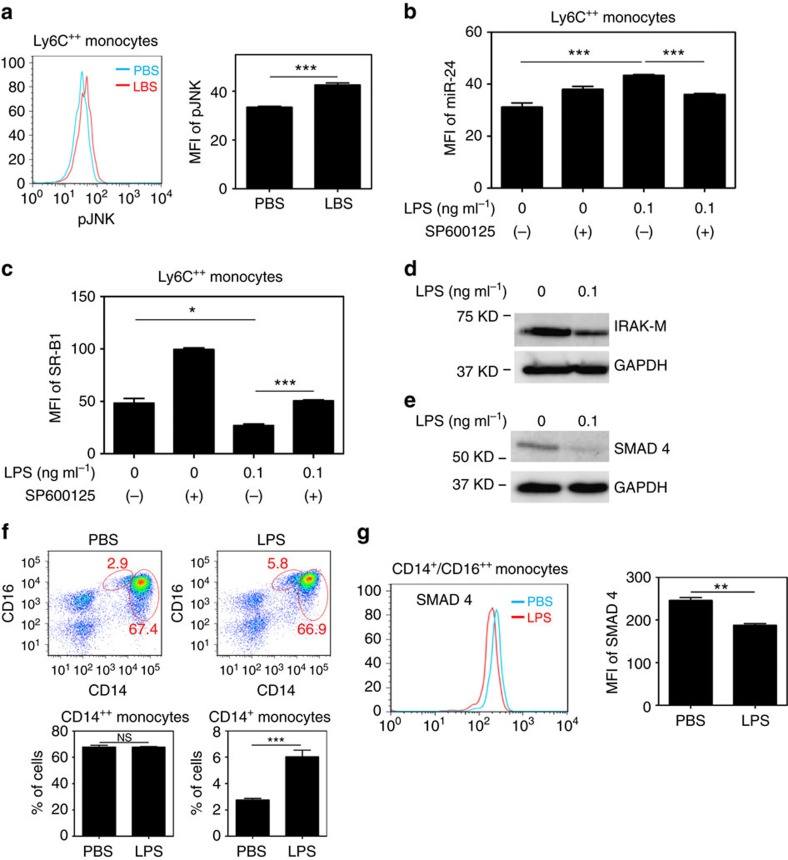
Polarization of low-grade inflammatory monocytes through the reduction of IRAK-M. (**a**–**e**) BMMs from C57 BL/6 mice were cultured with M-CSF (10 ng ml^−1^) in the presence LPS for 5 days and SP600125 was added to some cultures. Fresh LPS and SP600125 was added every 2 days. (**a**) JNK phosphorylation within CD11b^+^Ly6C^++^ monocytes treated with super-low-dose LPS (0.1 ng ml^−1^) was determined by flow cytometry. (**b**) Fluorescent RNA probe for miR-24-3p was added to the cell cultures 16 h before harvesting. The expression levels of miR-24-3p within CD11b^+^Ly6C^++^ monocytes were examined by flow cytometry. (**c**) The expression levels of SR-B1 within CD11b^+^Ly6C^++^ monocytes were examined by flow cytometry. (**d**,**e**) Western blot analyses of SMAD4 and IRAK-M expression in the cell cultures. (**f**,**g**) Peripheral blood mononuclear cells collected from healthy individuals were cultured with M-CSF (100 ng ml^−1^) in the presence super-low-dose LPS (5 pg ml^−1^) for 2 days. The frequency of inflammatory monocytes (**f**) and SMAD4 expression within CD14^+^CD16^+^ population (**g**) was determined by flow cytometry. Quantified data are shown from cells with indicated treatment (*n*=3) and results are representative of three experiments. Error bars show means±s.e.m.; NS, not significant; *P<0.05, **P<0.01 and ***, *P*<0.001; (**a**,**f**,**g**) Student's *t*-test; (**b**) one-way analysis of variance.

Despite the pathological relevance, molecular mechanisms responsible for the polarization of low-grade inflammatory monocytes are not well understood. A key limitation of existing *in vitro* mechanistic studies regarding monocyte polarization and/or activation is the short time course being examined^40^. To better study the sustained polarization of monocytes, we cultured BMMs with macrophage colony-stimulating factor (M-CSF) together with subclinical-dose LPS (100 pg ml^−1^) for 5 days. We observed that the prolonged challenge with subclinical dose LPS caused a sustained elevation of JNK phosphorylation in the polarized CD11b^+^Ly6C^++^ pro-inflammatory monocytes, as measured by flow cytometry analyses (Fig. 5a). We further tested whether the sustained JNK activation may be responsible for the elevated expression of miR-24 in polarized monocytes. Indeed, we demonstrated that the selective JNK inhibitor SP600125 potently inhibited the induction of miR-24 in the CD11b^+^Ly6C^++^ pro-inflammatory monocytes challenged by super-low-dose LPS (Fig. 5b). JNK inhibition also restored monocyte SR-B1 levels (Fig. 5c).

To sustain chronic JNK activation and low-grade inflammation, the removal of negative feedback regulators responsible for homeostasis are often necessary^11^,^12^. To this regard, IRAK-M is a critical negative feedback regulator of the innate immunity signalling pathway within monocytes^16^ and its induction through Smad4-mediated gene expression is partially responsible for homeostatic tolerance and bringing inflamed monocytes back to homeostasis^21^,^41^. On the other hand, IRAK-M-deficient monocytes/macrophages have elevated JNK activation and fail to develop endotoxin tolerance^13^,^16^. A stronger stimulation of TLR (Toll-like receptor) pathway through higher doses of LPS induces homeostatic tolerance within macrophages following an initial transient wave of inflammatory response^40^. In contrast, we previously reported that super-low levels of LPS fail to induce macrophage homeostatic tolerance and can reduce the cellular levels of IRAK-M^11^. Here we further tested whether the prolonged treatment with subclinical-dose LPS may sustain inflammatory monocyte polarization through suppressing the negative feedback regulator IRAK-M. We observed that BMMs cultured in the presence of super-low-dose LPS for 5 days had significantly reduced levels of IRAK-M (Fig. 5d). As the expression of IRAK-M was known to be under the control of Smad4 (ref. 21), we tested the levels of Smad4 in cultured BMMs. We observed reduced Smad4 levels in monocytes cultured in the presence of super-low-dose LPS as compared with control monocytes (Fig. 5e).

In humans, the intermediate CD14^+^CD16^+^ monocytes represent the inflammatory subset and have been implicated in the pathogenesis of various inflammatory diseases including atherosclerosis^42^,^43^. We asked whether the mechanistic observation in mice may also be reflected in human monocytes. We observed that super-low-dose LPS significantly expanded the population of the intermediate CD14^+^CD16^+^ monocytes (Fig. 5f). On the other hand, the levels of Smad4 within the population of CD14^+^CD16^+^ monocytes were significantly diminished following the challenge with super-low-dose LPS (Fig. 5g).

Based on these observations, we tested whether *in vivo* conditioning with super-low-dose LPS may modulate the levels of IRAK-M and subsequently lead to sustained inflammation. Based on immuno-histochemical staining, we found that HFD-fed ApoE-deficient mice conditioned with super-low-dose LPS had significantly lower levels IRAK-M within the plaque macrophages (Supplementary Fig. 5). Western blot analyses also revealed a reduction in IRAK-M protein levels in harvested splenocytes from HFD-fed *ApoE*^−/−^ mice conditioned with super-low-dose LPS as compared with mice conditioned with PBS (Supplementary Fig. 5).

### Reduction of IRAK-M is due to miR-24-mediated suppression of Smad4

**Figure 6:**
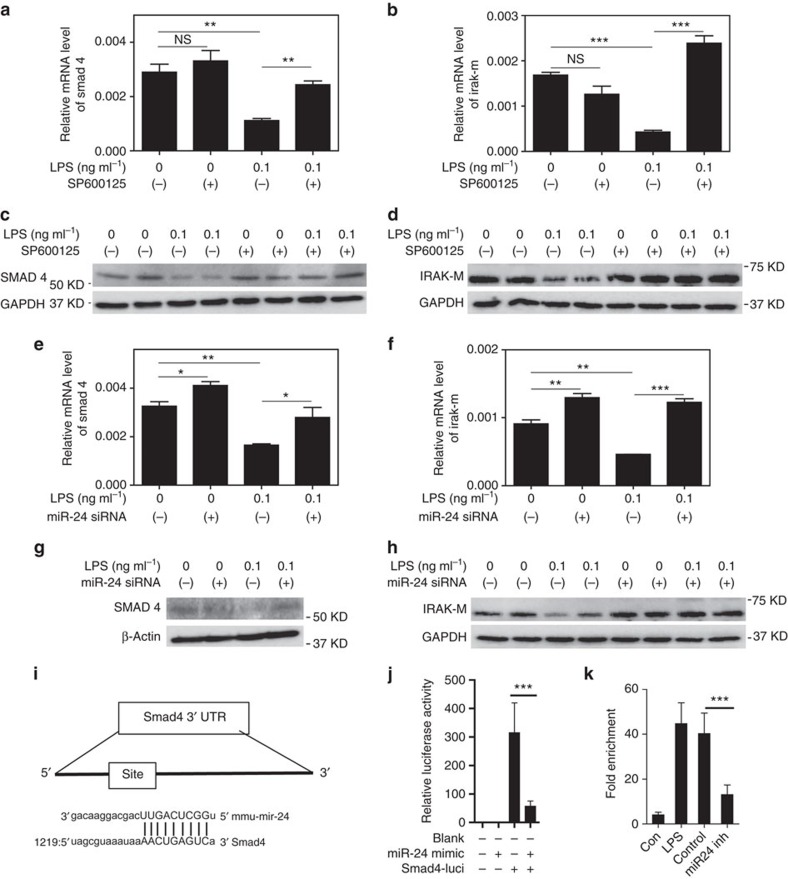
The disruption of the IRAK-M homeostatic circuit is due to miR-24-mediated suppression of Smad4. (**a**–**h**) BMMs from C57 BL/6 mice were cultured with M-CSF (10 ng ml^−1^) in the presence of LPS (0.1 ng ml^−1^) for 5 days and SP600125 or miR-24 antagomir was added to indicated cultures. Fresh LPS, SP600125 and miR-24 antagomir was added every 2 days. The mRNA and protein levels of SMAD4 and IRAK-M in the cell cultures were analysed by real-time reversre transcriptase–PCR and western blotting, respectively. (**i**) A schematic illustration of the SMAD4 3′-UTR and potential miR-24 binding sites. (**j**) Luciferase activity assays in 293T cells transfected with SMAD4 3′-UTR luciferase reporter plasmids in the presence of miR-24 mimic or the scramble control. (**k**) BMMs were cultured for 5 days and miR24 antagomir was transfected. On 7 days, the cells were stimulated by LPS (0.1 ng ml^−1^) for 4 h and then harvested for RNA co-immunoprecipitation (RIP). Quantified data are shown from cells with indicated treatment (*n*=3) and results are representative of three experiments. Error bars show means±s.e.m.; NS, not significant; *P<0.05, **P<0.01 and ***P<0.001; one-way analysis of variance.

Our data suggest that the negative feedback circuit of Smad4-IRAK-M responsible for monocyte tolerance and dampening of sustained JNK activation is compromised in low-grade inflammatory monocytes challenged with super-low-dose LPS. Next, we further tested whether super-low-dose LPS-mediated JNK activation may be responsible for the disruption of Smad4 and IRAK-M. Indeed, we found that the application of a selective JNK inhibitor blocked the reduction of Smad4 and IRAK-M in BMMs challenged by super-low-dose LPS (Fig. 6a–d). The alterations in protein levels of Smad4 and IRAK-M closely correlated with their mRNA levels (Fig. 6a–d), which suggest that the disruption of homeostatic Smad4 and IRAK-M was primarily due to reduced message levels. The administration of proteasome inhibitor MG-132 failed to restore the protein levels of Smad4 in BMMs challenged with super-low-dose LPS (Supplementary Fig. 6), lending further support for the modulation of Smad4 mRNAs during the disruption of monocyte tolerance and low-grade polarization.

To better understand the disruption of negative feedback circuitry, we aim to clearly define the molecular mechanism responsible for the reduction of Smad4 mRNA in low-grade inflammatory monocytes challenged with super-low-dose LPS. Given our above finding of sustained elevation of miR-24 mediated by chronic JNK activation, we tested whether elevated miR-24 may be critically responsible for the reduction of Smad4 mRNA and its downstream target IRAK-M. We observed that the application of miR-24 antagomir in cultured monocytes restored the RNA levels of both Smad4 and IRAK-M reduced by super-low-dose LPS (Fig. 6e,f). Likewise, the miR-24 antagomir also restored the protein levels of Smad4 and IRAK-M (Fig. 6g,h).

To test the mechanism for miR-24-mediated reduction of Smad4 and IRAK-M, we searched the 3′-UTR of Smad4 and IRAK-M, and found a highly conserved miR-24-binding site in the 3′-UTR of Smad4 (Fig. 6i). Luciferase reporter assay demonstrated that miR-24 mimic potently reduced the Smad4 mRNA stability (Fig. 6j). RNA co-immunoprecipitation analyses also showed direct association of miR-24 with the Smad4 3′-UTR within the microprocessor complex (Fig. 6k). Taken together, our data reveal that JNK-miR-24 directly contributes to the suppression of Smad4 expression, which leads to a subsequent reduction of homeostatic negative regulator IRAK-M, in low-grade inflammatory monocytes programmed by super-low-dose LPS.

### Disruption of IRAK-M polarizes pro-inflammatory monocytes

**Figure 7:**
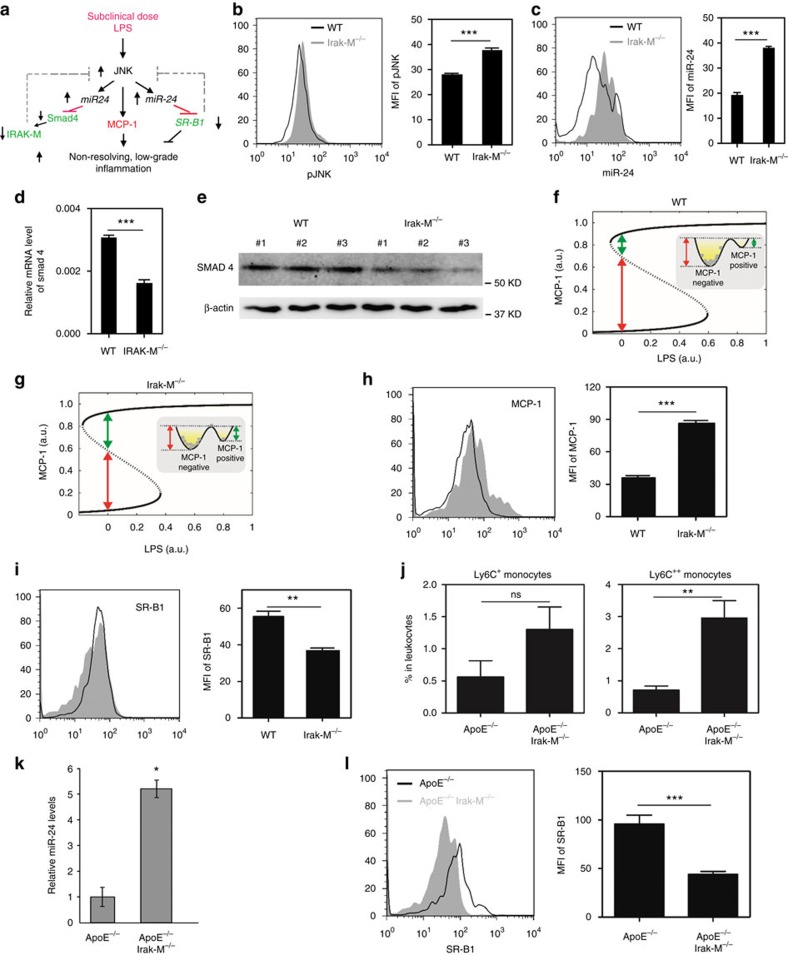
Disruption of IRAK-M results in sustained pro-inflammatory programming of monocytes both *in vivo* and *in vitro*. (**a**) A schematic illustration of network dynamics underlying the establishment of non-resolving inflammation. (**b**–**e**) BMMs from WT C57 BL/6 mice or *Irak-M*^−/−^ mice were cultured with M-CSF (10 ng ml^−1^) for 5 days. (**b**) JNK phosphorylation within CD11b^+^Ly6C^++^ monocytes. (**c**) The levels of miR-24-3p within CD11b^+^Ly6C^++^ monocytes . (**d**,**e**) The mRNA and protein levels of SMAD4. (**f**) The bifurcation diagram that illustrates a relatively tight suppression of MCP-1 expression in resting WT cells. Solid lines show two stable steady states (upper branch: MCP-1-positive state; lower branch: MCP-1-negative state). Dotted line is the unstable steady states. All cells reside at MCP-1-negative state because of the large energy barrier for a MCP-1-negative to MCP-1-positive state transition (red line). AU, conceptual arbitrary unit. (**g**) Deletion of IRAK-M allows leaky low-grade inflammation to occur as manifested in the leaky activation of MCP-1. Bifurcation diagram was plotted similar to (**f**). AU, arbitrary unit. (**h**) Flow cytometry analysis of MCP-1 levels within cultured CD11b^+^Ly6C^++^ monocytes from WT and *Irak-M*^*−/−*^ mice(*n*=3). (**i**) Expression levels of SR-B1 within cultured CD11b^+^Ly6C^++^ monocytes from WT and *Irak-M*^*−/−*^ mice (*n*=3). (**j**–**l**) *ApoE*^−/−^ and *ApoE*^−/−^/*Irak-M*^−/−^ mice were fed with HFD for 8 weeks. (**j**) Circulating CD11b^+^Ly6C^++^ and CD11b^+^Ly6C^+^ monocytes were examined by flow cytometry. Data are shown from *ApoE*^−/−^ (*n*=5) and *ApoE*^−/−^/*Irak-M*^−/−^ (*n*=6) mice. (**k**) The expression levels of miR-24 from splenocytes were analysed between *ApoE*^−/−^ (*n*=4) and *ApoE*^−/−^/*Irak-M*^−/−^ (*n*=4) mice. (**l**) The expression levels of SR-B1 within circulating monocytes were analyzed between *ApoE*^−/−^ (*n*=5) and *ApoE*^−/−^/*Irak-M*^−/−^ (*n*=6) mice. All data are representative of at least two similar experiments. Error bars show means±s.e.m.; NS, not significant; **P<0.01 and ***P<0.001; Student's *t*-test.

Our data map out an integrated negative feedback circuit that involves JNK-miR-24-mediated suppression of Smad4, which in turn leads to reduced expression of IRAK-M. The reduction of IRAK-M may allow sustained elevation of JNK and miR-24 in low-grade inflammatory monocytes programmed by super-low-dose LPS (Fig. 7a). Given the known role of IRAK-M in suppressing JNK activation and causing monocyte tolerance^13^, we plan to further confirm that the disruption of IRAK-M may lead to non-resolving inflammatory monocyte polarization through sustained miR-24 expression. Indeed, we observed elevated levels of pJNK and miR-24 in IRAK-M^−/−^ monocytes as compared with wild-type (WT) monocytes (Fig. 7b,c). Consequently, we observed reduced levels of Smad4 mRNA and protein in IRAK-M^−/−^ BMMs as compared with WT BMMs (Fig. 7d,e).

Our data delineate that the IRAK-M-mediated negative feedback circuit is disrupted during the low-grade inflammatory polarization of monocytes (Fig. 7a). Our computational analyses of the refined circuitry support the hypothesis that the negative feedback regulator IRAK-M may enable monocytes to maintain the resting non-inflammatory state (Fig. 7f). Consequently, the lack of IRAK-M may fail to restrain the ‘leaky' inflammation and allow the low-level expression of MCP-1 in the absence of LPS (Fig. 7g). The expression levels of CCR5 in IRAK-M-deficient monocytes were also significantly higher as compared with WT monocytes (Supplementary Fig. 7). Indeed, our experimental analyses with cultured BMMs corroborated with our computational hypothesis. Almost all resting WT BMMs stained negative for the expression of MCP-1 through flow cytometry analyses (Fig. 7h). In contrast, distinct populations of MCP-1-positive BMMs were evident in the resting IRAK-M-deficient BMMs (Fig. 7h). On the other hand, IRAK-M-deficient BMMs have reduced levels of SR-B1 as compared with WT BMMs (Fig. 7i).

We next studied the *in vivo* monocyte activation status and the levels of SR-B1 in IRAK-M-deficient mice and monocytes. HFD-fed *ApoE*^−/−^/*Irak-M*^−/−^ mice had significantly higher levels of the CD11b^+^Ly6C^++^ pro-inflammatory monocytes in circulating blood (Fig. 7j). The levels of miR-24 expressed in splenocytes of HFD-fed *ApoE*^−/−^/*Irak-M*^−/−^ mice were significantly higher as compared with the miR-24 levels expressed in splenocytes of HFD-fed ApoE^−/−^ mice (Fig. 7k). Correspondingly, the levels of SR-B1 within the blood monocyte population were significantly reduced in HFD-fed *Apo*E^−/−^/*Irak-M*^−/−^ mice as compared with HFD-fed ApoE^−/−^ mice (Fig. 7l). The bone marrow monocytes from HFD-fed *ApoE*^−/−^/*Irak-M*^−/−^ mice also had significantly reduced levels of SR-B1 as compared to HFD-fed *ApoE*^−/−^ mice (Supplementary Fig. 7).

### IRAK-M deficiency aggravates atherosclerosis

**Figure 8:**
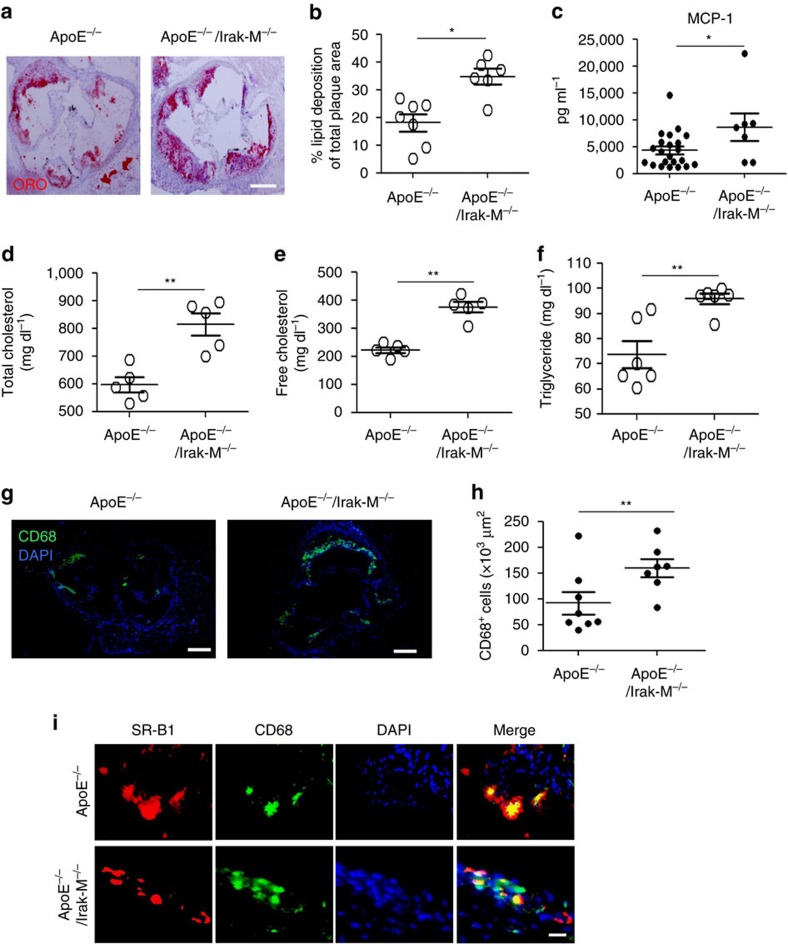
Lack of IRAK-M results in exacerbated atherosclerosis. *ApoE*^−/−^ and *ApoE*^−/−^/*Irak-M*^−/−^ mice were fed with HFD for 8 weeks. (**a**) Representative images of atherosclerotic plaques within aortic root areas stained by Oil Red O. Scale, 300 μm. (**b**) Quantification of the lipid deposition area as a percentage of the total atherosclerotic plaque area within the aortic root. Data are shown for aortic plaque areas from *ApoE*^−/−^ (*n*=7) and *ApoE*^−/−^/*Irak-M*^−/−^ (*n*=6) mice. (**c**) Levels of plasma MCP-1 from *ApoE*^−/−^ (*n*=22) and *ApoE*^−/−^/*Irak-M*^−/−^ (*n*=7) mice. Levels of total (**d**) and free cholesterol (**e**) in the plasma. Data are shown from *ApoE*^−/−^ (*n*=5) and *ApoE*^−/−^/*Irak-M*^−/−^ (*n*=5) mice. (**f**) Triglyceride levels in the plasma from *ApoE*^−/−^ (*n*=6) and *ApoE*^−/−^/*Irak-M*^−/−^ (*n*=6) mice. (**g**) Representative images of CD68^+^ macrophages in the atherosclerotic plaques of aortic root areas. Scale bar, 300 μm. (**h**) Quantification of CD68^+^ macrophages number per μm^2^ in the lesion area of aortic root. Data are shown for aortic plaque areas from *ApoE*^−/−^ (*n*=8) and *ApoE*^−/−^/*Irak-M*^−/−^ (*n*=7) mice. (**i**) Representative images of atherosclerotic plaques within aortic root areas co-stained with 4,6-diamidino-2-phenylindole (DAPI), as well as antibodies against SR-B1, CD68. Scale bar, 100 μm. All data are representative of two similar experiments. Error bars show means±s.e.m. *P<0.05 and **P<0.01; Student's *t*-test.

We next examined the progression of atherosclerosis in *ApoE*^−/−^/*Irak-M*^−/−^ mice. As compared with HFD-fed *ApoE*^−/−^ mice, HFD-fed *ApoE*^−/−^/*Irak-M*^−/−^ mice had significantly elevated levels of lipid deposition within the atherosclerotic plaques (Fig. 8a,b). Plasma levels of MCP-1, total cholesterol, free cholesterol and triglyceride were all significantly elevated in HFD-fed *ApoE*^−/−^/*Irak-M*^−/−^ mice as compared with these parameters in HFD-fed *ApoE*^−/−^ mice (Fig. 8c–f). Immunohistochemical staining revealed significantly higher levels of plaque macrophages in HFD-fed *ApoE*^−/−^/IRAK-M^−/−^ mice (Fig. 8g,h). As measured by real-time reversre transcriptase–PCR analyses, the levels of miR-24 were significantly elevated in splenocytes from HFD-fed *ApoE*^−/−^/*Irak-M*^−/−^ mice as compared with HFD-fed *ApoE*^−/−^ mice (Fig. 7k). Consequently, the levels of SR-B1 were dramatically reduced within plaque macrophages from HFD-fed *ApoE*^−/−^/*Irak-M*^−/−^ mice as compared with HFD-fed *ApoE*^−/−^ mice (Fig. 8i). In contrast, the levels of SR-A were significantly increased within plaque macrophages from FD-fed *ApoE*^−/−^/*Irak-M*^−/−^ mice as compared with HFD-fed *ApoE*^−/−^ mice (Supplementary Fig. 8).

## Discussion

Non-resolving inflammation is considered as a major contributor for chronic disease such as atherosclerosis. Despite extensive interest, the causes of non-resolving inflammation underlying atherosclerosis, however, have drawn debates with less clarity^4^. At first glance, the existence of persistent stimulatory signals coupled with prolonged and/or excessive responses seem to be the most intuitive answer and may appear partially consistent with observed phenomena^44^. However, there are ample instances that persistent inflammatory signals, when excessive, may trigger compensatory anti-inflammatory tolerance^45^,^46^. Thus, the sole emphasis on the presence of excessive positive signals may be an overly simplistic view and a far cry from the complex dynamics that underlies non-resolving inflammation as evident during the pathogenesis of atherosclerosis. Our current data support a concept that the dysfunction of a negative regulator may be critical for the establishment of non-resolving inflammation associated with the progression of atherosclerosis. We demonstrate that, under low-grade inflammatory condition with a subclinical level of endotoxemia, the negative suppressor of inflammation IRAK-M is significantly reduced both *in vitro* and *in vivo*. The reduced expression of IRAK-M in polarized inflammatory monocytes is achieved through JNK-mediated degradation of transcription factor SMAD4. As a consequence, the compromise in IRAK-M function allows a persistent low-grade activation of JNK, enables polarization of non-resolving inflammatory monocytes and progression of atherosclerosis.

To our knowledge, our study provides the first causal evidence that reveal the pathological significance of subclinical endotoxemia during atherosclerosis *in vivo*, a phenomenon in humans increasingly correlated with non-resolving inflammatory conditions^47^,^48^. Although previous attempts with LPS injection similarly led to increased atherosclerosis and related metabolic complications^9^,^49^, these studies used higher tolerant dosages of LPS (despite of often referred as ‘low dose' in the literature; >10 ng ml^−1^ through *in vitro* culture, >1 μg per mouse or >50 μg per kg body weight during *in vivo* studies). This is in sharp contrast to the pathologically relevant dosages of circulating plasma LPS observed in human patients or laboratory animals with metabolic endotoxemia (∼100 pg–1 ng ml^−1^ of plasma endotoxin, 100 pg–10 ng per mouse or 5 ng–0.5 μg per kg body weight through *in vivo* injection)^7^,^36^,^50^,^51^. The higher dosages of LPS are known to induce anti-inflammatory tolerance both *in vitro* and *in vivo*^52^,^53^, which is in distinct contrast to the low-grade pro-inflammatory polarization induced by pathologically relevant subclinical endotoxemia^40^. Endotoxin tolerance is manifest in monocytes with transient expression of pro-inflammatory cytokines, followed by a compensatory homeostatic ‘tolerant' state, through induction of multiple negative feedback regulators^14^,^54^. In addition, recent studies reveal that higher dosages of LPS can permeate inside cells and cause general cytotoxic effects through directly interacting with several caspases, independent of TLR4 (refs 32, 34). In contrast, we previously reported that super-low-dose LPS solely signals through cell surface TLR4 (ref. 11). Understandably, not aware of such complex LPS signalling dynamics, earlier studies with animal atherosclerosis models used higher dosages of LPS^9^,^49^. Thus, the effects of these studies on atherosclerosis are likely to be compounded by toxic effects of higher dose LPS unrelated to TLR4 and may not be truly reflective of low-grade inflammation induced by pathologically relevant dosages of endotoxemia. Our current study provides much needed clarity for studying fundamental mechanisms responsible for the establishment of non-resolving inflammation underlying atherosclerosis. Our previous *in vitro* studies indicate that innate monocytes/macrophages fail to develop tolerance when challenged with a subclinical super-low-dose LPS *in vitro*^11^,^12^. Extending these observations, our current study demonstrates that a prolonged stimulation with subclinical dose LPS can sustain the low-grade inflammatory polarization of monocytes both *in vitro* and *in vivo*. The polarized monocytes not only exhibit elevated inflammatory potential as reflected in the expression of MCP-1 and CCR5, but also have reduced ability for cholesterol homeostasis due to reduced expression of SR-B1. SR-B1 is a key molecule involved in cholesterol metabolism potentially through regulating lecithin-cholesterol acyltransferase activities and lipid transport^27^,^55^. The genetic deletion of SR-B1 has been shown to be critically involved in elevated plasma levels of cholesterol and elevated atherosclerosis as well^27^,^56^. Our data reveal that the reduction of SR-B1 and inflammatory monocyte polarization are critically coupled, due to the elevated expression of miR-24 by polarized inflammatory monocytes. Together, our data explain the aggravated atherosclerosis due to subclinical endotoxemia, characterized by low-grade non-resolving inflammation, elevated cholesterol levels in plasma and atherosclerotic plaques.

Our data extend the emerging concept of innate immune memory in a pathologically relevant model of non-resolving inflammation and atherosclerosis. Through adoptive transfer experiment, we demonstrated that programmed inflammatory monocytes by super-low-dose LPS are responsible for aggravated atherosclerosis. Recent studies suggest that innate immune leukocytes may be ‘trained' and adopt ‘memory' states with either reduced or increased inflammatory potential *in vitro*^54^,^57^. The development of innate memory, if well defined, may have significant impacts on our understanding of chronic inflammatory disease and may reconcile the varying disease severity in individuals with distinct innate immune environments. However, existing experimental systems regarding innate memory largely used *in vitro* cell culture systems with short incubation periods^40^,^57^. Our data reveal that chronic conditioning of mice *in vivo* with subclinical-dose LPS may cause a long-term polarization of inflammatory monocytes. Intriguingly, the polarization and memory effect can persist 1 month after the stoppage of LPS injection. Mechanisms that contribute to the development of innate leukocyte memory are poorly studied. Based on systems analyses of cellular memory in general, the establishment of distinct memory states may require the existence of dynamic circuits with competitive feedbacks^58^,^59^,^60^. The removal of negative suppressors might stabilize memory states and allow for sustained polarization, even in the absence of stimulators. Our data are consistent with this concept and reveal that the reduction in IRAK-M expression may be critically involved in the establishment of polarized inflammatory monocytes. Our data further reveal the molecular mechanism for sustained reduction of IRAK-M, due to miR-24-triggered degradation of the transcription factor Smad4. Our data unravel that subclinical super-low-dose LPS programmes the sustained elevation of pJNK and miR-24 levels, to enable the non-resolving low-grade polarization of monocytes, due to the disruption of the Smad4-IRAKM negative feedback circuit both *in vitro* and *in vivo*. We demonstrated that the reduction in IRAK-M is also coupled to the reduction of SR-B1, aided by sustained activation of JNK and expression of miR-24. Reduced SR-B1 not only compromises cholesterol transport and facilitates the progression of foamy atherosclerotic plaques, but may further compromise the control of inflammation. Potentially through modulating the activities of lecithin-cholesterol acyltransferase^27^,^61^, SR-B1 plays a key role in lipid transport and metabolism. In addition, SR-B1 was also known to suppress inflammatory signalling process in macrophages^26^,^62^. Deletion of SR-B1 in mice was shown to elevate atherosclerosis through exacerbating inflammation and/or plasma lipid^27^,^56^. Consistent with our current study, Tao *et al*.^63^ demonstrated that adoptive transfer of SR-B1-deficient bone marrow cells significantly increased atherosclerosis, plaque necrosis, as well as reduced content of plaque collagen. Our data complement these studies and further define molecular mechanisms responsible for the reduction of SR-B1 by super-low-dose LPS, through the upregulation of miR-24. The reduction of SR-B1 through elevated miR-24 may further facilitate the establishment of polarized inflammatory monocytes. It is interesting to note that miR-24 is also among the most highly expressed miRs in plasma samples from human atherosclerosis patients^38^.We realize that additional feedback mechanisms may also be involved, which may include complex interplay among lipid alteration and low-grade inflammation, and our current approach may fail to fully clarify the integrated circuitries that underlie the complex pathogenesis of atherosclerosis. However, our study serves as a key step to present at least the cardinal principle for the establishment of non-resolving low-grade inflammation, with a specific focus on the disruption of a cardinal negative feedback regulator IRAK-M mediated by sustained expression of miR-24. Future integrated analyses that combine computational and experimental approaches are warranted to fully decipher this complex dynamic.

Taken together, our study reveals a disruption of an integrated negative feedback circuit responsible for the sustained polarization of low-grade inflammatory monocytes by subclinical-dose LPS *in vitro* and *in vivo*, as well as its pathological relevance in the aggravation of atherosclerosis. Subclinical-dose endotoxin selectively induces miR-24 that causes the reduction of negative feedback modulators of inflammation such as Smad4-IRAK-M, which in turn leads to sustained low-level activation of JNK, miR-24 and reduced expression of SR-B1. Future strategies aimed at restoring this negative feedback circuit may hold potential in treating chronic low-grade inflammatory disease such as atherosclerosis.

## Methods

### Experimental animals

*ApoE*^−/−^ mice were purchased from the Jackson Laboratory. *ApoE*^−/−^/*Irak-M*^−/−^ mice were obtained by crossing *ApoE*^−/−^ mice with *Irak-M*^−/−^ mice provided by Dr Richard A. Flavell at Yale University School of Medicine. These mice were bred and maintained in the animal facility at Virginia Tech with the approved protocol from the Intuitional Animal Care and Use Committee in compliance with the US National Institutes of Health Guide for the Care and Use of Laboratory Animals. All experiments used male mice of 7–10 weeks when experiments were initiated. Animal numbers were empirically determined to consider the need for statistical significance and also adhere to Intuitional Animal Care and Use Committee policy on minimizing animal numbers. Breeding and handling limitations were also considered for determining the number of animals used for experiments. No randomization method was used due to the experimental setup. Animals that showed health concerns unrelated to the experimental conditions (for example, fight wounds and dermatitis) were excluded.

### HFD feeding and LPS injection

Two experimental models were employed in this study. In the first model, High Fat Western Diet (Harlan Teklad 94059)-fed mice were intraperitoneally injected with either PBS or LPS (5 ng per kg body weight) every 3 days for 2 months. In the second model, Western Diet (Harlan Teklad 94059)-fed mice were intraperitoneally injected with either PBS or LPS (5 ng per kg body weight) twice weekly for 1 month. Following the termination of injection, mice were continuously fed with Western Diet (Harlan Teklad 94059) for another month. Animals were monitored daily and body weights were taken weekly.

### Histology and immunofluorescence

Histological analyses were performed on fresh-frozen, optimal cutting temperature (OCT) compound-embedded proximal aortic sections (10 μm). Slides were fixed in 4% neutral buffered formalin for 5 min. Haematoxylin and eosin, as well as Oil Red O staining were performed. The total lesion area and the percentage of vessel occlusion were measured. The percentage of vessel occlusion was measured as the ratio of the vessel intima area (without plaque) to the vessel lumen area (with plaque). All calculations analysed used the mean of samples from six mice per group. Immunofluorescence analyses were performed on fresh-frozen, OCT-embedded proximal aortic sections (10 μm). Slides fixed in 4% neutral buffered formalin for 5 min were stained with anti-mouse primary antibodies, anti-mouse MOMA-2 (Santa Cruz, catalogue number: sc-59332, 1/100 dilution), SR-B1 (Novus, Littleton, CO, catalogue number: NB400-104, 1/100 dilution), SR-A (Bio-Rad, Hercules, CA, catalogue number: MCA1322PE, 1/100 dilution) and IRAKM (Santa Cruz, catalogue number: sc-366015, 1/100 dilution) antibodies, followed by a biotinylated anti-rabbit Ig secondary antibody (BD Biosciences, San Jose, CA, catalogue number: 562418, 1/100 dilution) or biotinylated anti-rat Ig secondary antibody (eBioscience, San Diego, CA, catalogue number: 13-4813-85, 1/100 dilution) and then Streptavidin-phycoerythrin (eBioscience, catalogue number: 12-4317-87, 1/100 dilution) or Streptavidin-fluorescein isothiocyanate (BioLegend, San Diego, CA; catalogue number: 405202, 1/100 dilution). 4,6-Diamidino-2-phenylindole was used to stain the nucleus.

### Lipid analyses

Plasma was collected from all mice at the time of killing. The total cholesterol and free cholesterol in the plasma were measured by using Cholesterol Quantitation Kit (Sigma-Aldrich, St Louis, MO). For the measurement of triglycerides, the Triglyceride Quantification Colorimetric/Fluorometric Kit (Biovision, Milpitas, CA) was used.

### ELISA of cytokines and chemokines

Plasma samples collected from peripheral blood were subjected to ELISA analyses for selected cytokines and chemokines. The kits for measurement of tumour necrosis factor-α, IL-10, IL-6, transforming growth factor-β and MCP-1 were from eBioscience.

### Adoptive transfer of *in vitro* cultured murine monocytes

Bone marrow cells isolated from WT C57 BL/6 mice or IRAK-M^−/−^ mice were cultured in RPMI 1640 medium supplemented with 10 % fetal bovine serum, 2 mM L-glutamine, 1% penicillin/streptomycin and with M-CSF (10 ng ml^−1^) in the presence of super-low-dose LPS (100 pg ml^−1^), and mirVana miR-24 antagomir (10 nM, Life Technologies, Carlsbad, CA) or JNK inhibitor SP600125 (10 μM, Sigma-Aldrich), was also added to the cell cultures in some experiments. Fresh LPS, miR-24 antagomir and SP600125 was added to the cell cultures every 2 days. After 5 days, cells were harvested and stained with anti-Ly6C (BioLegend, catalogue number: 128018, 1/200 dilution), anti-Ly6G (BioLegend, catalogue number: 127610, 1/200 dilution), anti-CD11b (BioLegend, catalogue number: 101226, 1/200 dilution), anti-CCR5 (BioLegend, catalogue number: 107006, 1/200 dilution) and anti-SR-B1 (Novus, catalogue number: NB400-131G, 1/400 dilution) antibodies. Stained cells were analysed by FACSCanto II and monocytes were gated within Ly6G-negative population. To test the expression of miR-24 in living cells, 100 pM SmartFlare RNA probe of miR-24-3p (Millipore, Billerica, MA) was added to the cell cultures and incubated at 37 °C for 16 h. The cells were harvested and stained with anti-Ly6C, anti-Ly6G and anti-CD11b antibodies. To determine the phosphorylation of JNK in inflammatory monocytes, cells were harvested after 5 days culture followed by fixation and permeabilization using Phosflow kit (BD Biosciences) and then stained anti-pJNK (T183/Y185, BD Biosciences, catalogue number: 562418, 1/100 dilution), anti-Ly6C, anti-Ly6G and anti-CD11b antibodies. To determine the production of MCP-1, BMMs cultured for 5 days were treated with phorbol myristate acetate (20 ng ml^−1^), ionomycin (1 μg ml^−1^) and GolgiStop protein transport inhibitor (BD Biosciences) for 4 h, and then stained with anti-Ly6C, anti-Ly6G and anti-CD11b antibodies. After fixation and permeabilization using Cytofix/Cytoperm kit (BD Biosciences), cells were stained with anti-MCP-1 antibody (BioLegend, catalogue number: 505904, 1/100 dilution). Mean fluorescent intensity of miR-24 probe, phosphorylation of JNK and production of MCP-1 within the CD11b^+^/Ly6G^-^/Ly6C^+^ inflammatory monocytes were determined by flow cytometry. The data were processed by FACSDiva or Flow Jo. For the adoptive transfer study, bone marrow cells isolated from *ApoE*^−/−^ mice were cultured in RPMI 1640 medium supplemented with 10 % fetal bovine serum, 2 mM L-glutamine, 1% penicillin/streptomycin and with M-CSF (10 ng ml^−1^) in the presence of either subclinical super-low-dose LPS (100 pg ml^−1^) or PBS for 5 days. Cells were washed three times with LPS-free sterile PBS and suspended in sterile PBS for injection. HFD-fed *ApoE*^−/−^ mice (8 weeks old, male) were transfused once weekly through intravenous injection with 3 × 10^6^ cells suspended in 200 μl sterile PBS for 4 weeks. One week after the last transfer regimen, mice were harvested for subsequent analyses.

### *In vitro* culture of human monocytes and flow cytometry analyses

Peripheral blood collected from healthy individuals was purchased from Research Blood Components, LLC (Boston, MA). Peripheral blood mononuclear cells were isolated using Histopaque-1119 and Histopaque-1077 (Sigma-Aldrich), and then cultured with M-CSF (100 ng ml^−1^) in the presence of super-low-dose LPS (5 pg ml^−1^). After 2 days, cells were harvested and stained with anti-CD14 (BioLegend, catalogue number: 325618, 1/100 dilution) and anti-CD16 antibodies (BioLegend, catalogue number: 302006, 1/100 dilution). After fixation and permeabilization using transcription factor buffer set (BD Biosciences), cells were stained with anti-SMAD 4 antibody (R&D Systems, catalogue number: IC2097P, 1/100 dilution). The samples were then analysed by FACSCanto II (BD Biosciences). The data were processed by or Flow Jo (Tree Star, Ashland, OR).

### *In vivo* analysis of monocyte phenotype

Peripheral blood and bone marrow cells were isolated from mice treated as described. Single-cell suspensions from the aorta were also prepared according to the protocol described before^64^. Cells were stained with anti-Ly6C, anti-Ly6G, anti-CD11b and anti-CCR5 antibodies (BioLegend), and anti-SR-B1 antibody (Novus). The samples were then analysed by FACSCanto II (BD Biosciences) and monocytes were gated within Ly6G-negative population. The data were processed by FACSDiva (BD Biosciences) or Flow Jo (Tree Star).

### Real-time reverse transcriptase–PCR analyses

Total RNA was isolated with QIAzol lysis reagent (Qiagen, Valencia, CA). First strand of complementary DNA was synthesized using a reverse transcription kit (Applied Biosystems, Foster City, CA) and real-time PCR was performed using SsoAdvanced SYBR green supermix (Bio-Rad) on the CFX96 real-time PCR instrument (Bio-Rad). The PCR cycles involve 3 min denaturation at 95 °C for 3 min, followed by 40 cycles of 95 °C for 15 s and 60 °C for 60 s. The relative levels of different transcripts were calculated using the ΔΔCt method and results were normalized based on the expression of β-actin within the same experimental setting. Primers were synthesized by Integrated DNA Technologies (Coralville, IA) and sequences are as follows: mouse *Irak-M* 5′-ACTAGAGCTGGCTGCATATTTC-3′ (sense), 5′-GTGCCATTTGTGCACTGTAATC-3′ (antisense); mouse *β-actin*, 5′-TTTCCAGCCTTCCTTCTTGG-3′ (sense), 5′-GGCATAGAGGTCTTTACGGATG-3′ (antisense). Relative expression of *Irak-M* was normalized to the expression of *β-actin*, which served as an internal control.

### Immunoblotting

BMMs isolated from C57 BL/6 mice were cultured as described before^31^. On day 5, total cell lysate was extracted with RIPA buffer (Thermo Scientific, Waltham, MA) containing a protease inhibitor cocktail (Sigma-Aldrich). Protein was applied to SDS–PAGE and transferred to a polyvinylidene difluoride membrane. The membrane was blocked and incubated with primary rabbit anti-IRAK-M (Santa Cruz, catalogue number: sc-366015, 1/100 dilution), anti-SMAD 4 (Cell Signaling, catalogue number: 9515S, 1/100 dilution), anti-GAPDH (Cell Signaling, catalogue number: 5174S, 1/100 dilution) or β-actin antibody (Cell Signaling, catalogue number: 4970S, 1/100 dilution) overnight at 4 °C, followed by incubation with horseradish peroxidase -linked anti-rabbit IgG secondary antibody (Cell Signaling, catalogue number: 7074S, 1/500 dilution) for 1 h at room temperature. Blots were developed by a chemiluminescence ECL detection kit (Thermo Scientific). Original uncropped blots are presented in Supplementary Figs 9,10 and 11.

### Construction of 3′-UTR plasmid constructs and luciferase reporter assay

The mouse full-length 3′-UTR of SR-B1 (NM_016741.1) or IRAK3 (NM_028679.3) containing miR-24 potential binding sites was amplified by PCR using following primers: *Sr-B1* (forward) 5′-cccaagcttggg CCCGCTTCTTGAGGACTCTC-3′, *Sr-B1* (reverse) 5′-aaaagtactttt GCCTCCGAATACCCTCTGGT-3′; *Smad4* (forward) 5′-ctagactagtctag GCCCTAACCATTTCCAGGAT-3′, *Smad4* (reverse) 5′-cccaagcttggg TACTGCCACCTTGCAGAACA-3′; *Irak3* (forward) 5′-cccaagcttggg TCCCCGTGAAAAGACACGAG-3′, *Irak3* (reverse) 5′-aaaagtactttt ATGCCACAGGCAACTCTTCT-3′. The PCR product was subcloned into a psiCHECK-2 vector (Promega) immediately downstream to the luciferase gene sequence. A pMIR-REPORT vector construct containing 3′-UTR of vascular endothelial growth factor with a mutant seed sequence of miR-24 was also synthesized using the primers: mut *Sr-B1* (forward): 5′-GTGACATCTGGGGATCTAGGCCATGGCACTGGTGGGCTG-3′, mut *Sr-B1* (reverse): 5′-CAGCCCACCAGTGCCATGGCCTAGATCCCCAGATGTCAC-3′. All constructs were verified by DNA sequencing. HEK293 cells were plated in 24-well clusters, then co-transfected with 500 ng constructs with or without miR-24 mimic. At 40 h after transfection, luciferase activity was detected using a dual-luciferase reporter assay system (Promega) and normalized to *Renilla* activity.

### miRNAseq analyses

Bone marrow monocytes from WT mice were cultured with complete RPMI medium and treated with PBS or 5 pg ml^−1^ LPS for 4 h. miRNAs were harvested with the miRNeasy kit from Qiagen according to the manufacturer's protocol. miRNAseq library were constructed using Illumina TruSeq Small kit. Constructed libraries were sequenced on HiSeq 1000 machine. Sequence reads were first trimmed for adaptor and checked for quality using a combination of custom perl scripts and Btrim64 software. Trimmed fastq sequences were imported into mirAnalyzer for mapping, miRNA assembly and quantification.

### Computational modelling analyses

We intend to build a simple and abstract mathematical model to illustrate the role of IRAK-M in preventing leaky low-grade inflammation, based on experimental evidence from this study as well as previous studies^65^,^66^. We posit that sustained and low levels of JNK activities potentiate MCP-1 expression and non-resolving low-degree inflammation. Low levels of JNK triggered by subclinical endotoxemia inhibits the expression of IRAK-M, which in turn inhibits the activation of JNK. The expression of miR-24 due to sustained JNK activation may lead to a suppression of SR-B1, which in turn can potentially suppress JNK (ref). Similar to refs 66, 67, 68, a formalism that allows capturing complex dependencies in a simple manner was used to translate the wiring diagram in Fig. 7a into ordinary differential equations. In these equations (equations 1, 2, 3, 4, 5, 6, 7), the levels of JNK (represented by *X*), SR-B1 (represented by *Y*), IRAK-M and MCP-1 on a logarithm scale (with a base of 10) are represented by italicized variables (*X*, *Y*, *IRAKM* and *MCP1*, respectively). We assume that all these variables have a dynamical range of tenfold; thus, they vary between 0 and 1. The parameter *γ*_*i*_ determines the rates at which specie *i* (*i*=*X*, *Y* and *IRAKM*) approaches its time-varying ‘target' value, 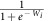, which is a sigmoidal function varying between 0 and 1, with a value of ½ when *W*_*i=*_0. *W*_*i*_ is the net activation or inhibition on *i* and its leading component, *ω*_*i*_, determines whether *i* is activated or inhibited when there are no regulatory signals impinging on *i* from any species in the motif. *ω*_*i,j*_ is the strength of the influence any specie *j* to *i* in the motif (*ω*_*ij*_> 0 for activation and <0 for inhibition). The arbitrary levels of LPS are represented by arbitrary units with italicized parameter *LPS*. For simplicity, we assume the arbitrary level of MCP-1 is same as the regulator *X* (JNK). The model parameters listed in Supplementary Table 2 were manually adjusted to qualitatively fit the experimental observations. XPP-AUT 5.91 (http://www.math.pitt.edu/bard/xpp/xpp.html, Department of Mathematics, the University of Pittsburgh) was used to build the model and generate bifurcation diagrams.


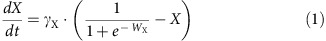



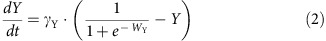



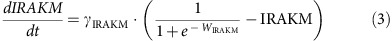



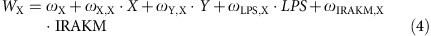



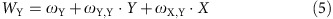



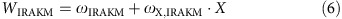



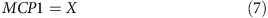


### Statistical analysis

Statistical analysis was performed with Prism software (GraphPad Software, La Jolla, CA). Values were expressed as means±s.e.m. The significance of the differences was assessed by Student's *t*-test or one-way analysis of variance where appropriate. *P*<0.05 was considered statistically significant.

### Data availability

The miRNAseq data have been deposited to the NCBI GEO databank with an accession number GSE87396. All relevant data are available from the corresponding authors upon request.

## Additional information

**How to cite this article:** Geng, S. *et al*. The persistence of low-grade inflammatory monocytes contributes to aggravated atherosclerosis. *Nat. Commun.* **7,** 13436 doi: 10.1038/ncomms13436 (2016).

**Publisher's note:** Springer Nature remains neutral with regard to jurisdictional claims in published maps and institutional affiliations.

## Supplementary Material

Supplementary Information Supplementary Figures 1-11 and Supplementary Table 1-2.
