# Supplementary material for: The persistence of low-grade inflammatory monocytes contributes to aggravated atherosclerosis
Source: Nat Commun. 2016 Nov 8;7:13436. doi: 10.1038/ncomms13436 (PMC5105176; doi:10.1038/ncomms13436)
Supplement: Supplementary Information — Supplementary Figures 1-11 and Supplementary Table 1-2. [file ncomms13436-s1.pdf]

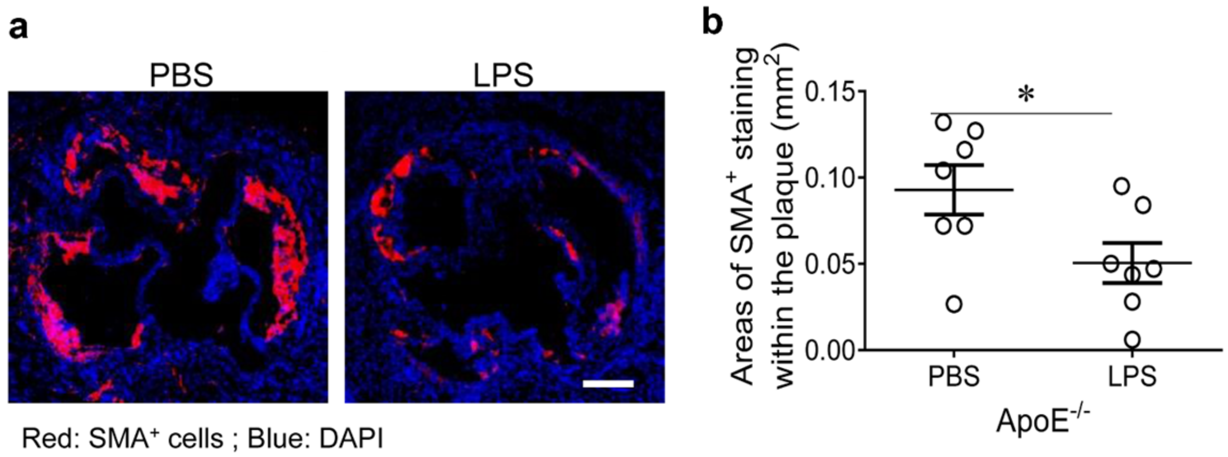

Supplementary Fig 1. Subclinical endotoxemia reduces plaque smooth muscle actin (SMA) levels. ApoE$^{-/-}$ mice were pre-conditioned with PBS or super-low dose LPS for 4 weeks together with high fat diet, followed by high fat diet feeding only for an additional 4 weeks. **(a)** Representative images of SMA^{+} staining within atherosclerotic plaques of aortic root areas. Scale bar: 300 µm. (**b**) Quantification of SMA^{+} positive staining areas per mm^{2} in the lesion area of aortic root. Data are shown for aortic plaque areas from PBS (n = 7) and super-low dose LPS conditioned (n = 7) mice. Data represent two similar experiments. Error bars show means ± s.e.m.; * $P < 0.05$; student t-test.

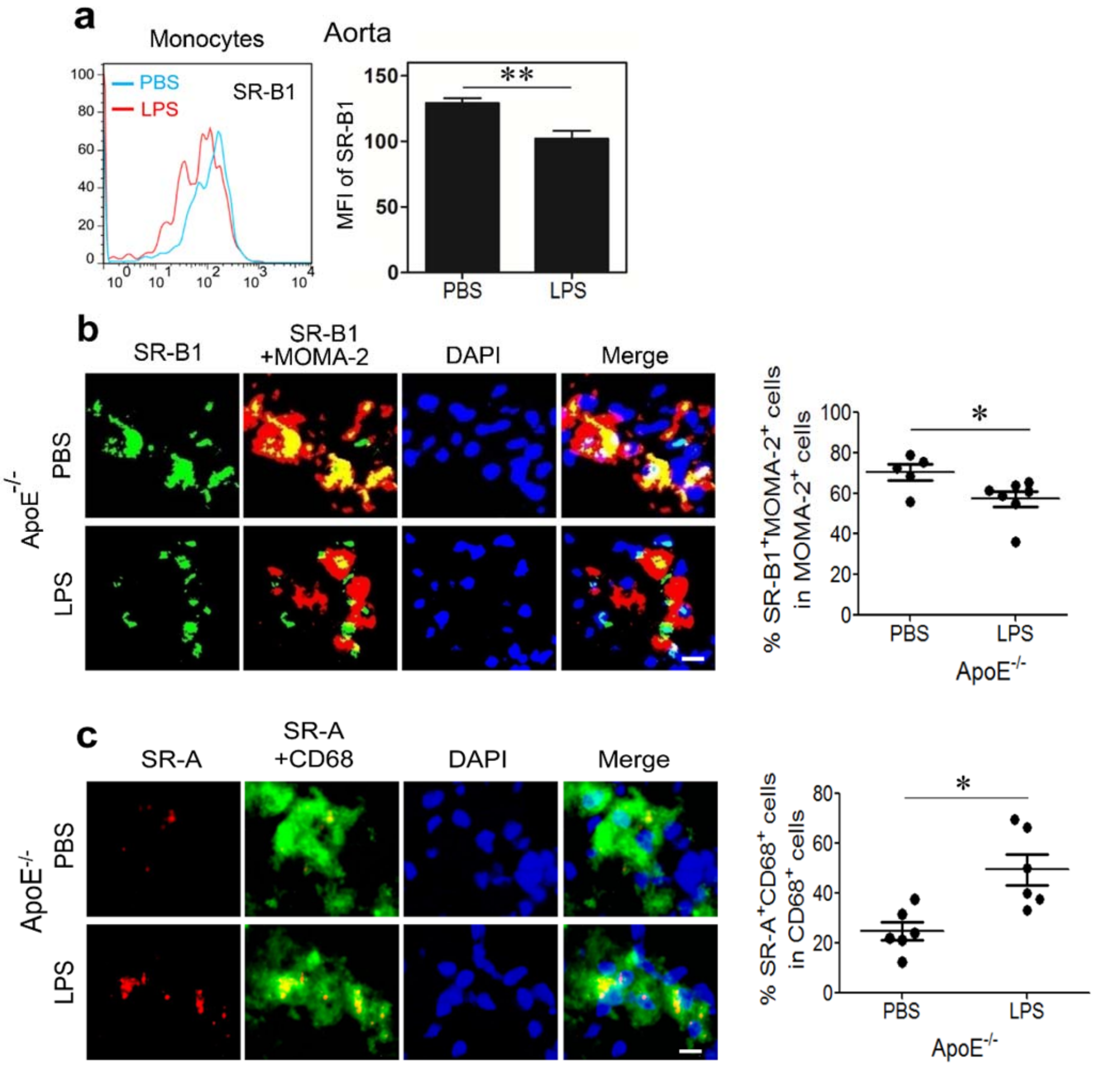

Supplementary Fig 2. Subclinical endotoxemia increases the expression of SR-A and reduces the expression of SR-B1. *$ApoE^{-/-}$* mice were pre-conditioned with PBS or super-low dose LPS for 4 weeks together with high fat diet, followed by high fat diet feeding only for an additional 4 weeks. (**a**) Single cell suspensions were prepared from aorta, and the expression levels of SR-B1 within monocytes were also analyzed. Data are shown for aortic monocytes from PBS (n = 6) and super-low dose LPS conditioned (n = 6) mice. (**b**) Representative images of SR-B1$^+$MOMA-2$^+$ macrophages in the atherosclerotic plaques of aortic root areas. Scale bar: 100 µm. Quantification of SR-B1$^+$MOMA-2$^+$ area as a percentage of the total atherosclerotic plaque area within the aortic root. (**c**) Representative images of atherosclerotic plaques within aortic root areas after staining with anti-SR-A and anti-CD68 antibodies. Scale bar: 100 µm. The percentage of SRA$^+$ cells within CD68$^+$ macrophages was quantified. Data represent two similar experiments. Error bars show means ± s.e.m.; * $P < 0.05$; ** $P < 0.01$; student t-test.

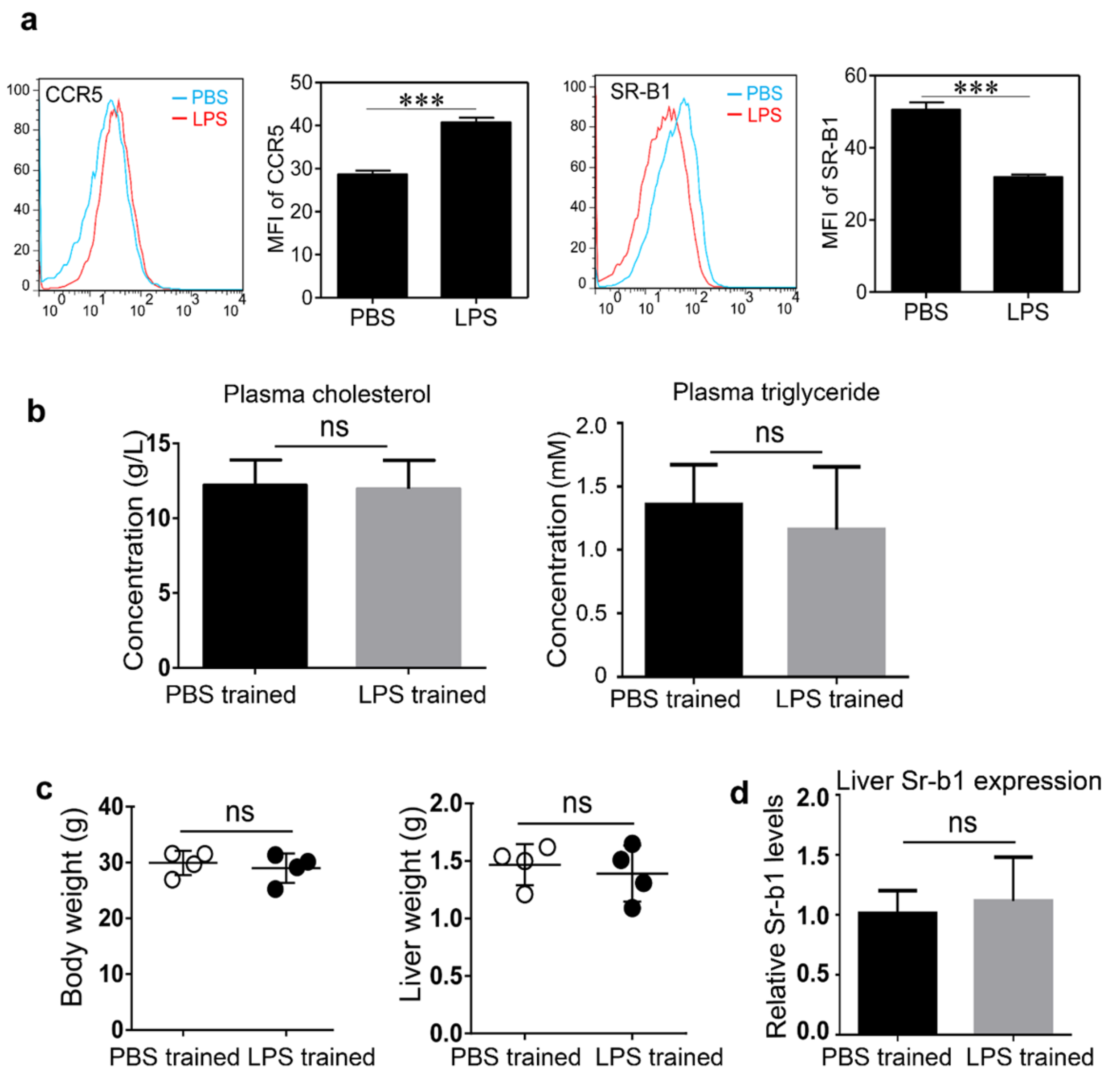

Supplementary Fig 3. Adoptive transfer of monocytes programmed by super-low dose LPS does not alter plasma levels of lipids or overall body and liver weights. BM cells from *ApoE*$^{-/-}$ mice were cultured with M-CSF (10 ng/ml) in the presence of either PBS or LPS (0.1 ng/ml) for 5 days. Surface expression levels of CCR5 and SR-B1 on inflammatory monocytes were determined by flow cytometry. (**b-c**) PBS or LPS programmed (trained) BM cells were then adoptively transferred through *i.v.* injection to HFD-fed ApoE $^{-/-}$ mice (3×10^6 cells/mouse) once a week for 4 weeks. (**b**) Plasma levels of total cholesterol and triglyceride were measured and quantified. (**c**) Total body weight and liver weight were quantified. (**d**) Liver levels of Sr-b1 expression were measured by real-time RT-PCR. Data represent two similar experiments. Error bars represent means ± s.e.m.; ns: no significance, ***, $P < 0.001$, student t-test.

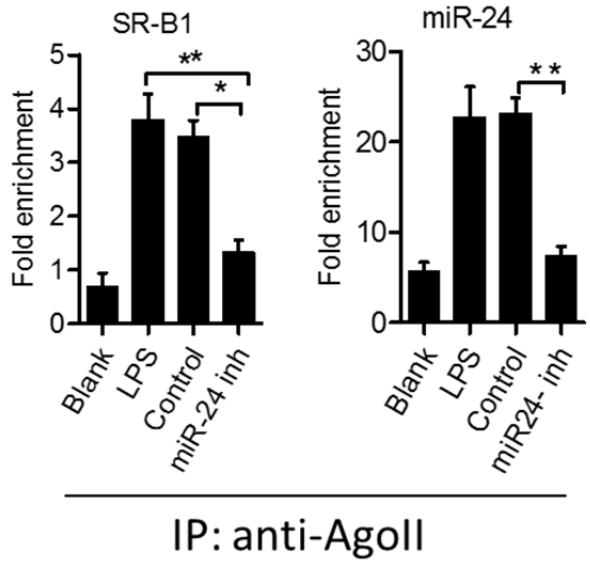

Supplementary Fig 4. miR-24 forms a close complex with Sr-b1 mRNA as measured by the RIP assay. Total RNAs were harvested from LPS treated WT bone marrow macrophages (30×10^6) and used for co-immunoprecipitation with an anti-AgoII antibody, in the presence of either miR-24 mimics or miR-24 antagomoir . The relative enrichment of Sr-b1 messenger RNAs were analyzed by real-time RT-PCR. Results are presentative of three experiments and are expressed as fold enrichment relative to AgoII-immunoprecipitation control samples.

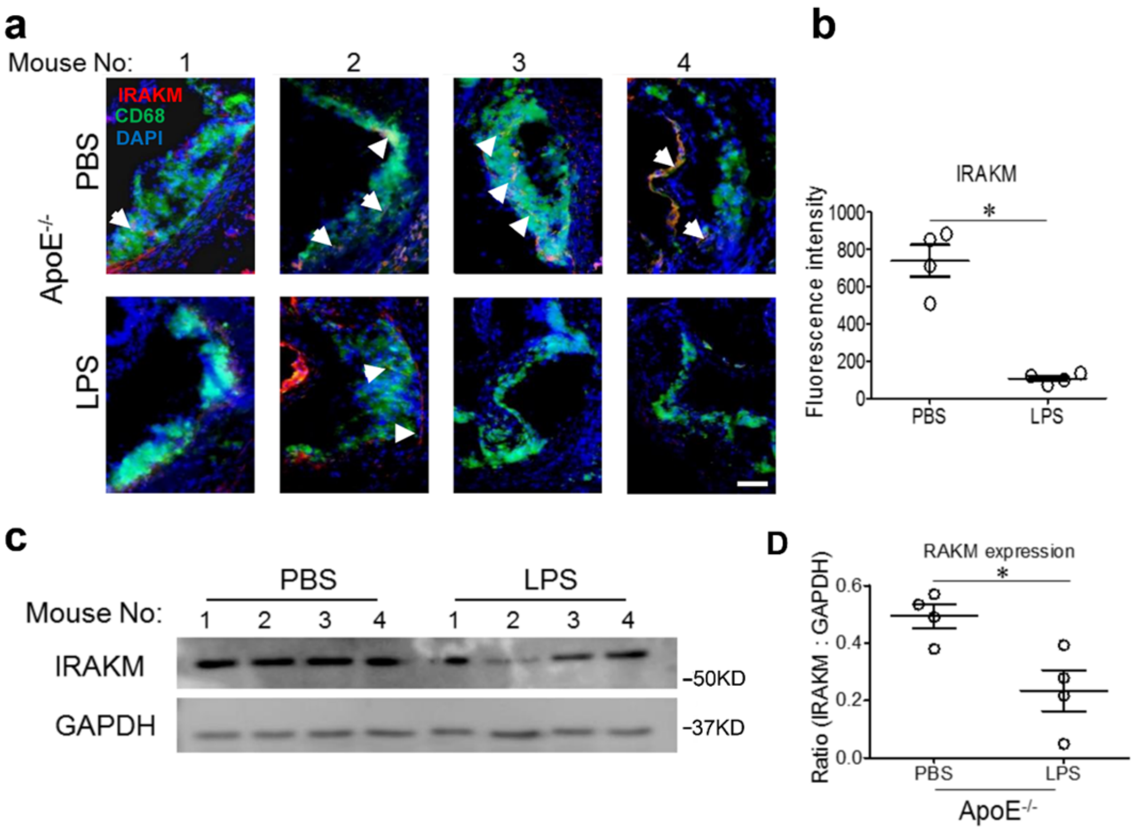

Supplementary Fig 5. Reduced IRAK-M expression in mice with subclinical endotoxemia. ApoE$^{-/-}$ mice were pre-conditioned with PBS or super-low dose LPS for 4 weeks together with high fat diet, followed by high fat diet feeding only for an additional 4 weeks. (**a**) Representative images of atherosclerotic plaques within aortic root areas after staining with anti-IRAK-M and anti-CD68 antibodies. Scale bar: 100 μm. (**b**) The fluorescent intensity of IRAK-M was quantified. Data are shown from PBS (n = 4) and super-low dose LPS conditioned (n = 4) mice. (**c**) Western blot analyses of IRAK-M expression in the splenic cells. (D) Relative expression levels of IRAK-M were quantified. Data are shown from PBS (n = 4) and super-low dose LPS conditioned (n = 4) mice. Results are presentative of two experiments. Error bars show means ± s.e.m.; *, $P < 0.05$; student t-test.

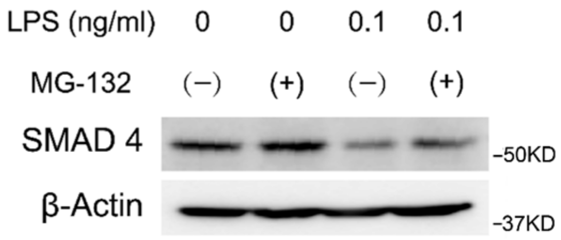

Supplementary Fig 6. The reduction of Smad4 in monocytes by super-low dose LPS is not caused by protein degradation. WT BMM were cultured in the presence of absence of 100 pg/ml LPS, together with or without MG-132 (25 nM) for 5 days. The cellular levels of Smad4 were detected by Western Blot. Data represents two similar experiments.

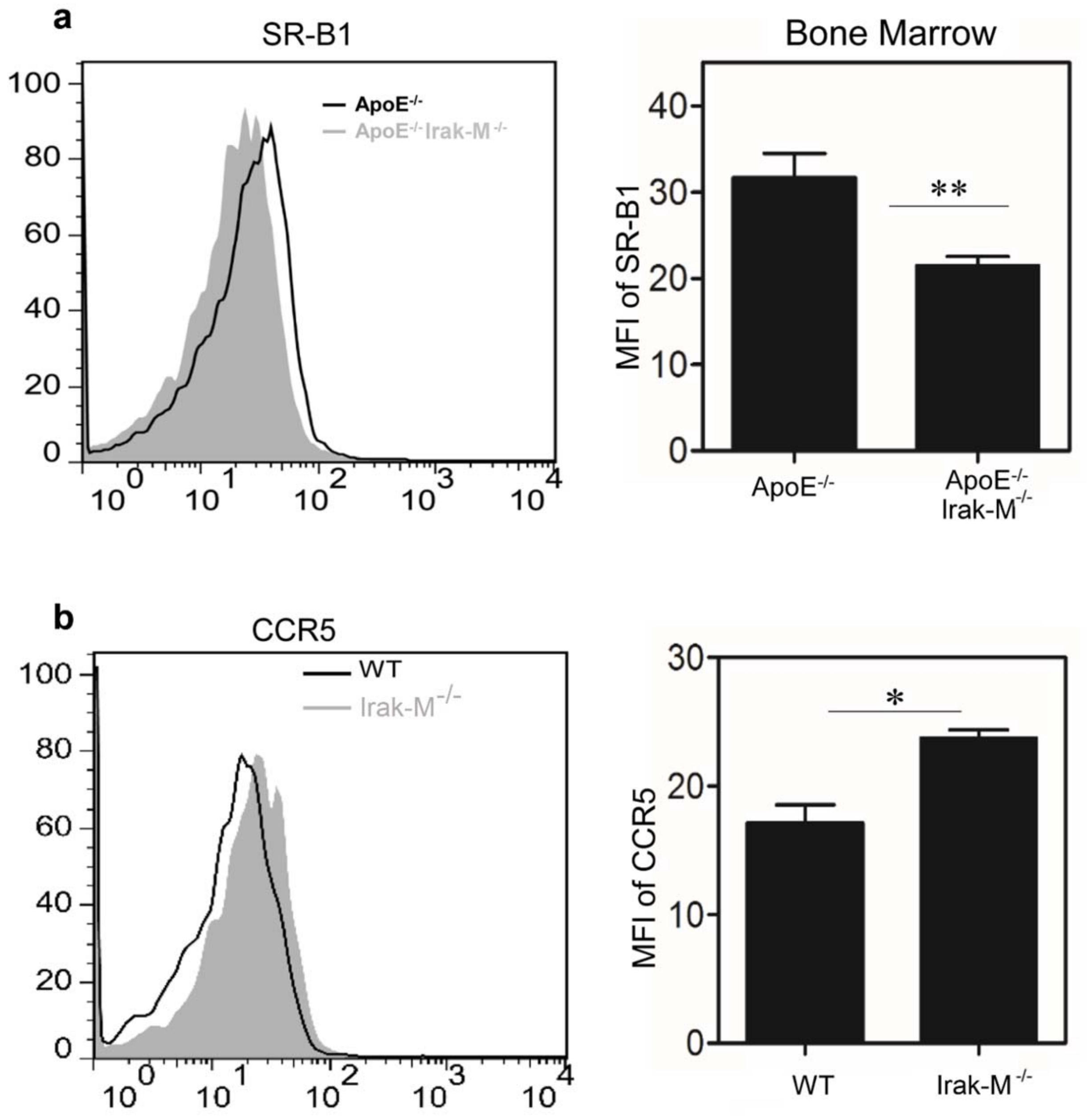

Supplementary Fig 7. Disruption of IRAK-M suppresses SR-B1 and up-regulates CCR5. (a) *ApoE*$^{-/-}$ and *ApoE*$^{-/-}$/*Irak-M*$^{-/-}$ mice were fed with HFD for 8 weeks. The expression levels of SR-B1 within BM monocytes were compared between *ApoE*$^{-/-}$ (n = 7) and *ApoE*$^{-/-}$/*Irak-M*$^{-/-}$ (n = 6) mice. Error bars show means ± s.e.m.; **, P < 0.01; student t-test. (**b**) BM cells from WT C57 BL/6 mice or *Irak-M*$^{-/-}$ mice were cultured with M-CSF (10 ng/ml) in the presence of super-low dose LPS (0.1 ng/ml). On day 5, the expression levels of CCR5 within CD11b^{+}Ly6C^{++} monocytes were examined. Quantified data are shown from WT and *Irak-M*$^{-/-}$ cells (n = 3). Results are representative of three experiments. Error bars show means ± s.e.m.; *, P < 0.05; student t-test.

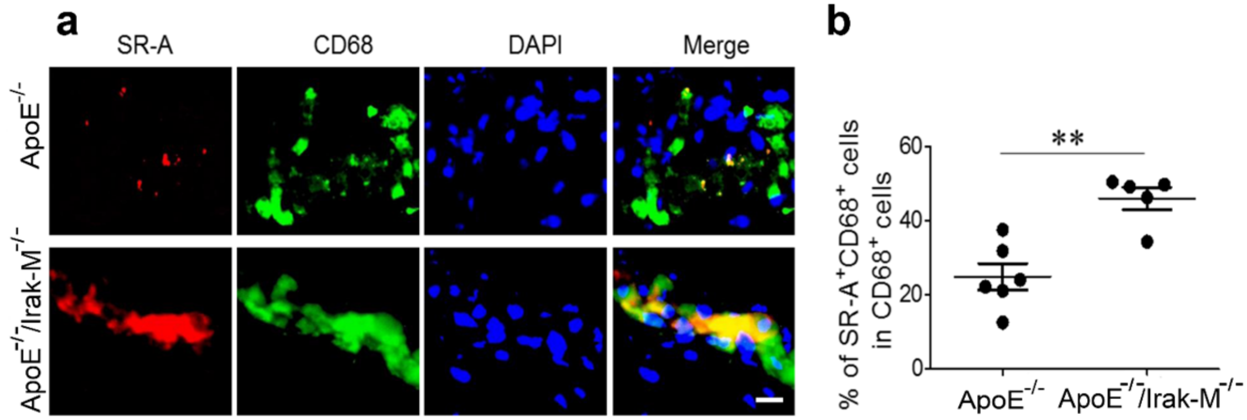

Supplementary Fig 8. Disruption of IRAK-M increases SR-A expression of macrophages within lesion area. *ApoE*$^{-/-}$ and *ApoE*$^{-/-}$/*Irak-M*$^{-/-}$ mice were fed with HFD for 8 weeks. (**a**) Representative images of atherosclerotic plaques within aortic root areas after staining with anti-SRA and anti-CD68 antibodies. Scale bar: 100 μm.(**b**) The percentage of SRA^{+} cells within CD68^{+} macrophages was quantified. Data are shown from *ApoE*$^{-/-}$ (n = 6) and *ApoE*$^{-/-}$/*Irak-M*$^{-/-}$ (n = 5) mice. Results are representative of two similar experiments. Error bars show means ± s.e.m.; ** $P < 0.01$; student t-test.

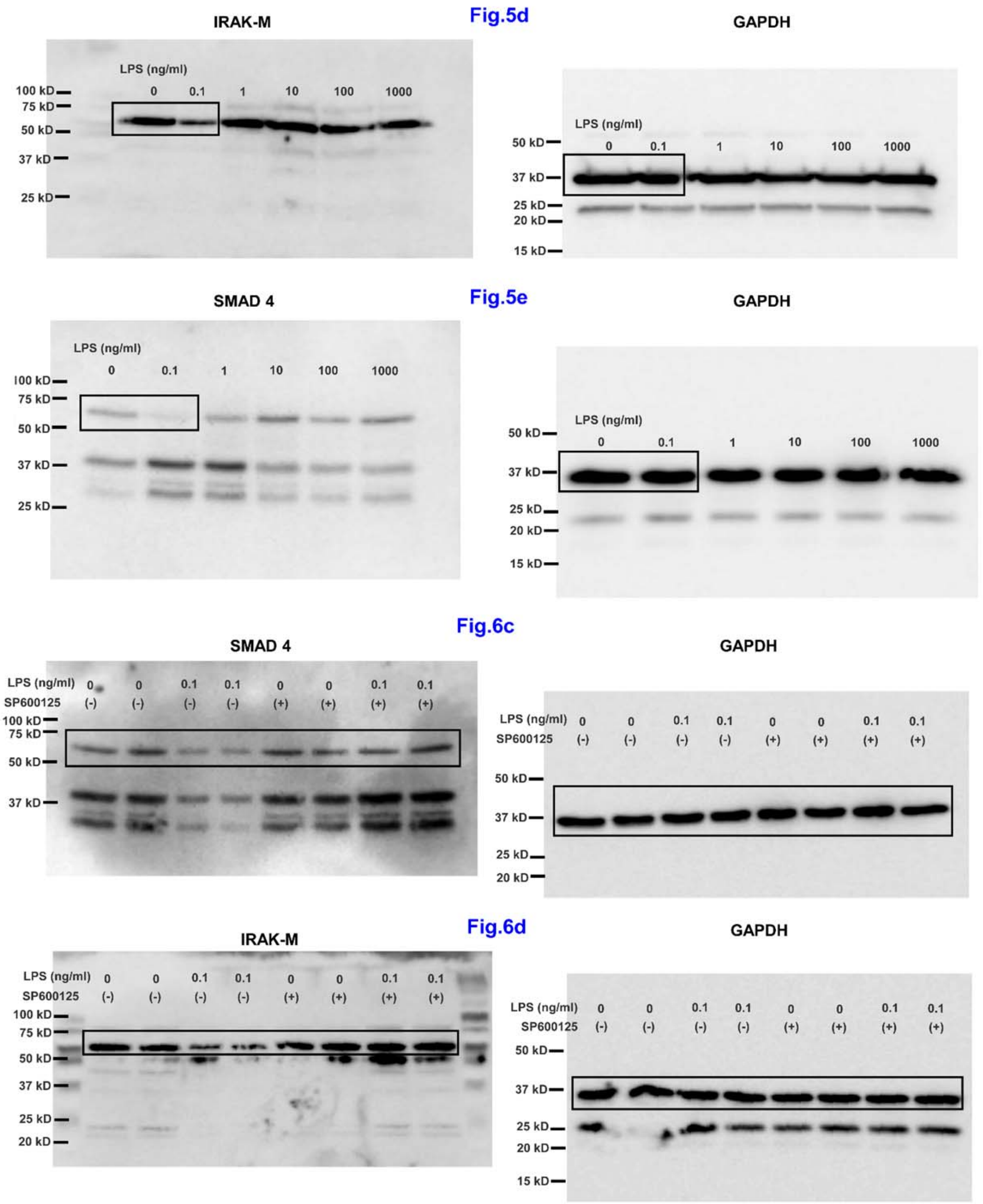

Supplementary Fig 9. Original gels shown in the main manuscript. Blots correspond to those shown in Figure 5d, 5e, and Figure 6c, and 6d within the main manuscript.

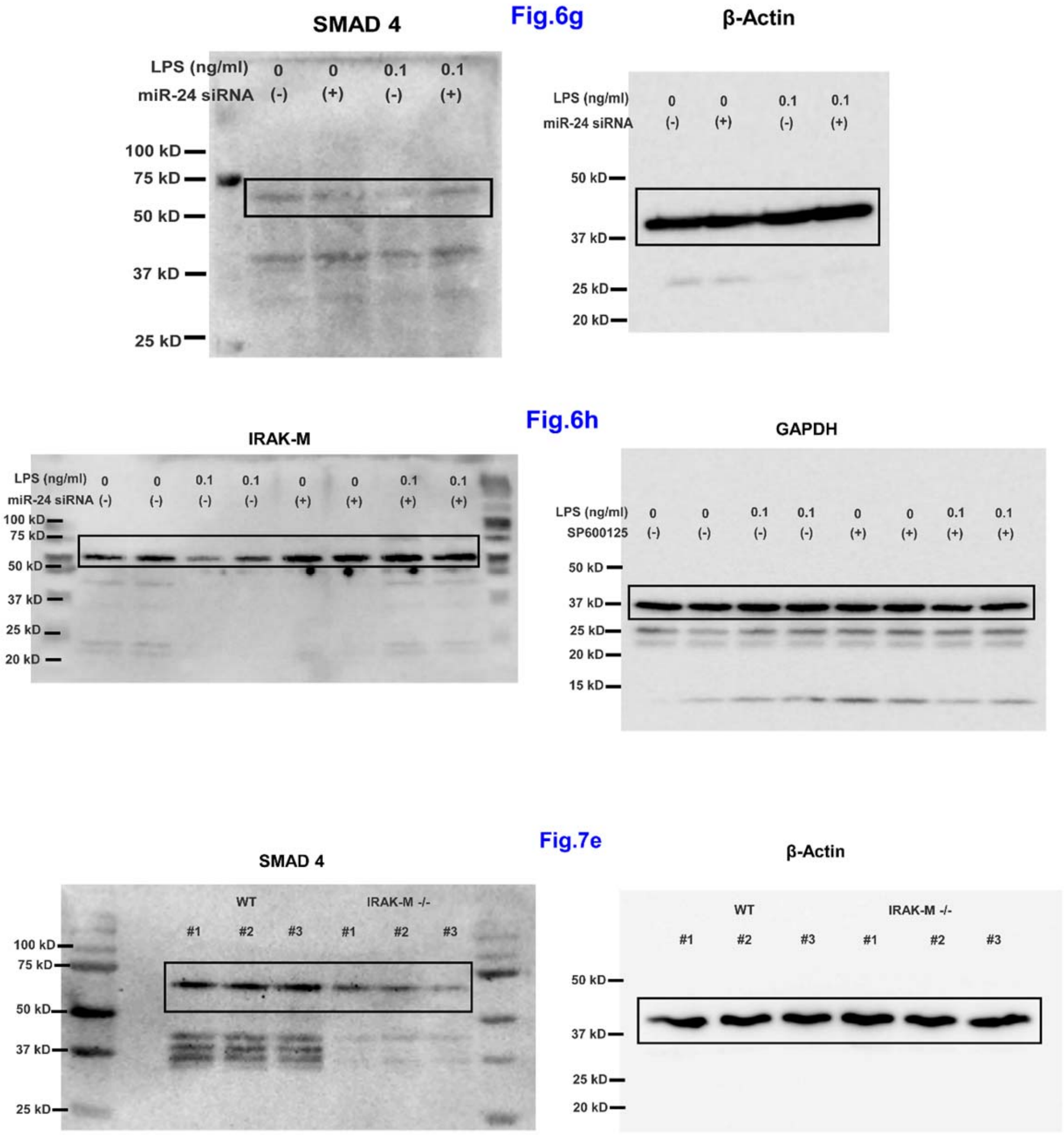

Supplementary Fig 10. Uncropped original gels shown in the main manuscript. Blots correspond to those shown in Figure 6g, 6h, and Figure 7f within the main manuscript.

Supplementary Fig. 5 c

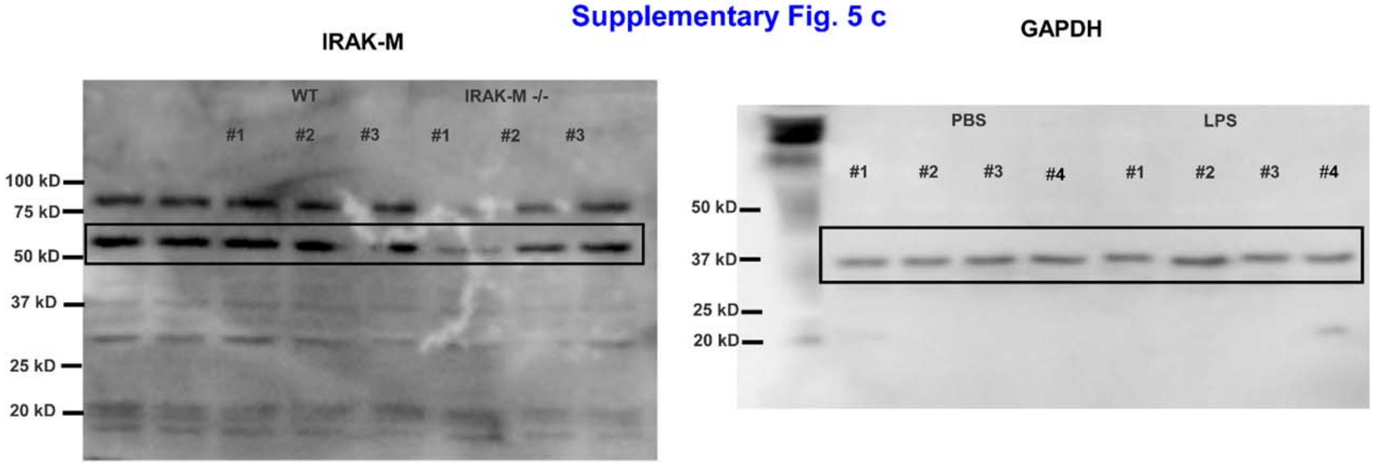

Supplementary Fig. 6

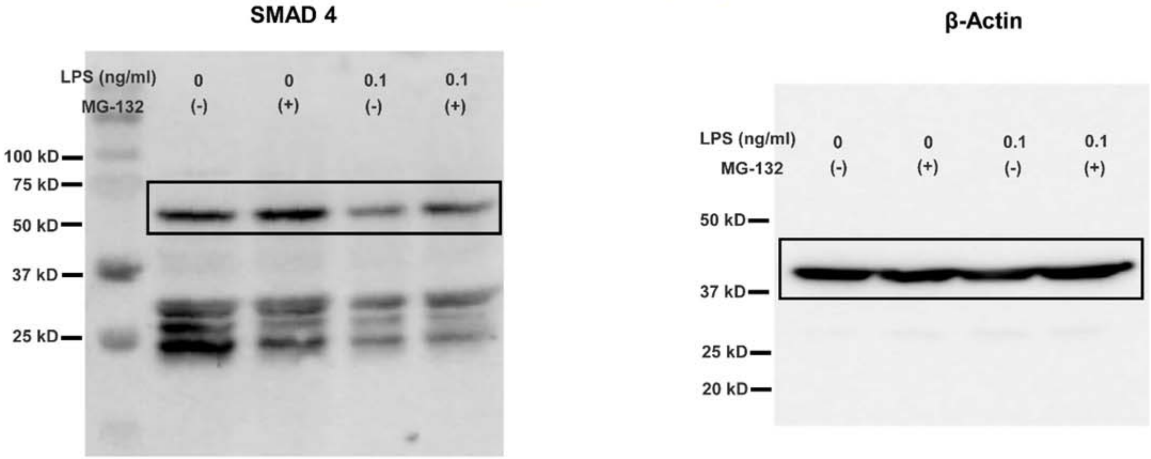

Supplementary Fig 11. Uncropped original gels shown in the Supplementary Fig 5c and Supplementary Fig 6

Supplementary Table 1. miRs selectively induced by subclinical dose LPS

id	PBS	LPS	Fold Change	log2Fold Change
mmu-miR-24-3p	5163.2669	57785.0508	11.19156766	3.4843402
mmu-miR-3068-5p	19.061420	183.523741	9.628020362	3.2672391
mmu-miR-146a-5p	6615.9765	55275.4873	8.35484932	3.0626138
mmu-miR-1965	0.7773613	6.05221449	7.78558712	2.9608058
mmu-miR-1953	0.9549579	5.23425630	5.481137857	2.4544754
mmu-miR-450b-3p	3.4718013	17.9982757	5.184131734	2.3741023
mmu-miR-449a-5p	2.1102558	9.17771225	4.349099244	2.1207166
mmu-miR-152-3p	359.01571	964.823028	2.687411723	1.4262173
mmu-miR-6236	11.600608	30.2133288	2.604460776	1.3809847
mmu-miR-29b-2-5p	16.226452	41.4310964	2.553305899	1.3523663
mmu-miR-200a-3p	22.1733757	55.1966714	2.489321973	1.31575284
mmu-miR-6240	218.527628	471.464116	2.157457709	1.10933228
mmu-miR-2137	37.9225004	75.8060019	1.998971613	0.99925798
mmu-miR-155-5p	186.703613	337.736411	1.808944158	0.85514787
mmu-miR-188-5p	329.895138	594.127773	1.800959471	0.84876571
mmu-miR-29a-5p	112.545596	201.605426	1.79132221	0.84102486
mmu-miR-5115	251.576054	445.134329	1.769382744	0.82324615
mmu-miR-194-5p	132.146298	230.562523	1.744752032	0.80302201

Supplementary Table 2. Arbitrary Model Parameter Values

Parameter	Description	Value
γ_X	Rate of *X* reaching its steady state	1
γ_Y	Rate of *Y* reaching its steady state	1
γ_{IRAKM}	Rate of *IRAK-M* reaching its steady state	1
ω_X	Basal inhibition of X	−1.4
$\omega_{X,X}$	X auto-activation	6.4
$\omega_{Y,X}$	X inhibition by Y	-2
$\omega_{LPS,X}$	X activation by LPS	1
$\omega_{IRAKM,X}$	X inhibition by IRAK-M	-1
ω_Y	Basal activation of Y	0.2
$\omega_{Y,Y}$	Y auto-activation	6.4
$\omega_{X,Y}$	Y inhibition by X	-2
ω_{IRAKM}	Basal activation of IRAK-M	0.2
$\omega_{X,IRAKM}$	IRAKM inhibition by X	-2
LPS	Level of LPS	0 ~ 1
